# Effect of replacement of soybean oil by *Hermetia illucens* fat on performance, digestibility, cecal microbiome, liver transcriptome and liver and plasma lipidomes of broilers

**DOI:** 10.1186/s40104-023-00831-6

**Published:** 2023-03-01

**Authors:** Lea Schäfer, Sarah M. Grundmann, Garima Maheshwari, Marcus Höring, Gerhard Liebisch, Erika Most, Klaus Eder, Robert Ringseis

**Affiliations:** 1 Institute of Animal Nutrition and Nutrition Physiology, Justus Liebig University Giessen, Heinrich-Buff-Ring 26-32, 35392 Giessen, Germany; 2 Institute of Nutritional and Food Sciences, Molecular Food Technology, University of Bonn, Friedrich-Hirzebruch-Allee 7, 53115 Bonn, Germany; 3 Institute of Clinical Chemistry and Laboratory Medicine, University Hospital of Regensburg, Franz-Josef-Strauss-Allee 11, 93053 Regensburg, Germany

**Keywords:** Broilers, Cecal microbiota, *Hermetia illucens*, Insect fat, Liver lipidome, Medium-chain fatty acids

## Abstract

**Background:**

In contrast to protein-rich insect meal, the feed potential of insect fat is generally less explored and knowledge about the suitability of insect fat as a fat source specifically in broiler diets is still limited. In view of this, the present study aimed to comprehensively investigate the effect of partial (50%) and complete replacement of soybean oil with insect fat from Hermetia illucens (HI) larvae in broiler diets on performance, fat digestibility, cecal microbiome, liver transcriptome and liver and plasma lipidomes. Thus, 100 male, 1-day-old Cobb 500 broilers were randomly assigned to three groups and fed three different diets with either 0 (group HI-0, *n* = 30), 2.5% (group HI-2.5, *n* = 35) or 5.0% (HI-5.0, *n *= 35) *Hermetia illucens* (HI) larvae fat for 35 d.

**Results:**

Body weight gain, final body weight, feed intake, and feed:gain ratio during the whole period and apparent ileal digestibility coefficient for ether extract were not different between groups. Cecal microbial diversity did not differ between groups and taxonomic analysis revealed differences in the abundance of only four low-abundance bacterial taxa among groups; the abundances of phylum Actinobacteriota, class Coriobacteriia, order Coriobacteriales and family Eggerthellaceae were lower in group HI-5.0 compared to group HI-2.5 (*P* < 0.05). Concentrations of total and individual short-chain fatty acids in the cecal digesta were not different between the three groups. Liver transcriptomics revealed a total of 55 and 25 transcripts to be differentially expressed between groups HI-5.0 vs. HI-0 and groups HI-2.5 vs. HI-0, respectively (*P* < 0.05). The concentrations of most lipid classes, with the exception of phosphatidylethanolamine, phosphatidylglycerol and lysophosphatidylcholine in the liver and cholesterylester and ceramide in plasma (*P *< 0.05), and of the sum of all lipid classes were not different between groups.

**Conclusions:**

Partial and complete replacement of soybean oil with HI larvae fat in broiler diets had no effect on growth performance and only modest, but no adverse effects on the cecal microbiome and the metabolic health of broilers. This suggests that HI larvae fat can be used as an alternative fat source in broiler diets, thereby, making broiler production more sustainable.

**Supplementary Information:**

The online version contains supplementary material available at 10.1186/s40104-023-00831-6.

## Introduction

In recent years, insect biomass has been increasingly recognized as an alternative and sustainable source of feed for monogastric livestock [1–3]. Consequently, the use of processed insect biomass as a feed for poultry and pigs has been authorized by the European commission in 2021 [4], with the aim of improving sustainability of food systems and securing food supply. Owing to its high protein content (40%–50% of dry matter (DM) depending on the insect species [5] and provided that the rearing substrate is suitable [6, 7]), research dealing with the feed potential of insect biomass has primarily focused on its role as a source of protein. In this context, a large number of studies in different monogastric livestock species demonstrated that insect larvae meal obtained from different edible insect species suitable for large-scale production, such as *Tenebrio molitor* (TM) and *Hermetia illucens* (HI), can replace conventional protein sources, like soybean meal, without impairing performance, metabolic health or product quality [8–11]. Apart from protein, insect larvae contain a significant amount of fat (up to 47% and 43% of DM in HI and TM, respectively, depending on the rearing substrate [12]), which can be obtained by a defatting process during insect meal production in order to increase the protein content and improve the storage stability of the insect meal. In contrast to insect meal, the feed potential of insect fat is far less explored. Despite that several studies have demonstrated that TM or HI fat has no negative impact on performance, gut morphology, selected blood parameters and product quality in broilers, laying hens and turkeys [13–21], knowledge about the suitability of insect fat as a fat source in broiler diets is still limited. In particular, in-depth analysis of the effects of insect fat on the gut microbiome and intermediary metabolism is lacking.

Despite that fatty acid composition of insect larvae is influenced by the fatty acids in the rearing substrate [5], the fatty acid composition of TM and HI fat are completely different. While TM fat consists mainly of unsaturated fatty acids (up to 80%), most of which are long-chain fatty acids (LCFA; C18:1 and C18:2; [11]), the majority of total fatty acids in HI fat are saturated fatty acids (SFA) [5, 16]. This explains that HI fat, but not TM fat has a hard consistency at room temperature. A further characteristic of HI fat is its high proportion of the medium chain-fatty acid (MCFA) lauric acid (C12:0) (≈ 40%; [16]). Interestingly, MCFA including lauric acid have been reported to exert antimicrobial effects (e.g., against Gram-positive cocci and *Escherichia coli*) in the gut of broilers [22, 23], and, thus, dietary inclusion of HI fat might alter the gut microbiota composition. Considering recent evidence that feed efficiency in broilers is affected by the gut microbiota composition through different mechanisms, such as a more complete digestion of substrates, an increased production of short-chain fatty acids (SCFA) or a decreased stimulation of the intestinal immune system [24, 25], an alteration of the gut microbiota might be of great relevance to broiler´s performance. In addition, convincing evidence exists that the gut microbiota has also a profound influence on energy metabolism and feeding behavior of the host due to the ability of the gut microbiota to communicate with the host along the gut-liver axis via different gut-derived compounds [26]. Moreover, MCFA-rich dietary fats might also exert a direct effect on energy metabolism, because triglyceride (TG)-bound MCFA are considered to be more efficiently absorbed than TG-bound LCFA due to easier emulsification and less dependence on pancreatic lipase [27, 28], thereby increasing digestible energy intake to support a higher growth performance. Against this background, the present study aimed to comprehensively investigate the effect of partial (50%) and complete replacement of soybean oil, the most commonly used fat source in commercial broiler diets, with HI larvae fat in broiler diets on performance, fat digestibility, cecal microbiome, liver transcriptome and liver and plasma lipidomes.

## Methods

### Animals and diets

The 35-d feeding trial was approved by the Animal Welfare Officer of the Justus Liebig University Giessen (approval no.: JLU 786_M). All experimental procedures described followed established guidelines for the care and handling of laboratory animals. The experiment included 100 male, 1-day-old broiler chickens (Cobb 500, Cobb, Wiedemar, Germany), which were randomly assigned to three groups (5 broilers/cage, group 1: 6 cages, group 2 and group 3: 7 cages). The mean initial body weight (BW) (44.9 ± 2.4 g; mean ± SD) was similar across the groups. The broilers were kept in 2.1 m^2^ cages equipped with nipple drinkers and feed automates and had free access to feed and water. At the floor of the cages there were cardboards, which were covered with litter to allow scratching, pecking and dustbathing. Cardboards and litter were exchanged 2 times per week during the first two weeks and every 2 d during the last three weeks of the trial. In addition, broilers were provided with perches in elevated position for resting and sleeping. Light intensity was constantly at 40 Lux and the light regime was 24 h:0 h, 23 h:1 h, 22 h:2 h, 21 h:3 h, 20 h:4 h, 19 h:5 h (light:dark) at d 1, 2, 3, 4, 5, 6, and 18 h:6 h from d 7 onward, as recommended by the breeder [29]. The room temperature decreased from 28–29 °C on d 1, measured at pen height, to 23–24 °C on d 35. During the first 6 d, infrared lamps (Albert Kerbl GmbH, Buchbach, Germany) were used as additional heat sources in order to adjust the temperature at the cage floor to 34 °C. Mean relative humidity was 60.0% ± 1.9%. The groups (group 1: HI-0, group 2: HI-2.5, group 3: HI-5.0) were fed three different diets, which varied only in the fat source (HI-0: 0 HI fat and 5% soybean oil, HI-2.5: 2.5% HI fat and 2.5% soybean oil, HI-5.0: 5.0% HI fat and 0 soybean oil), in a three-phase feeding system (starter diet form d 1 to 10, grower diet from d 11 to 21, finisher diet from d 22 to 35). The HI fat was purchased from Madebymade (Pegau, Germany) and stored at −20 °C until diet preparation. Prior to diet preparation, the stability of HI fat was assessed by determining the acid value and the percentage of polar compounds [30]. Low values for both parameters indicated no significant oxidation of the HI fat. Company’s details on the rearing conditions (substrate, duration) and further processing of the HI larvae are not available for reasons of confidentiality. The composition of the three diets are shown in Table 1. The finisher diets contained 0.5% titanium dioxide as indicator in order to calculate the apparent ileal digestibility (AID) coefficient for ether extract by the indicator method [31]. The diets met the broiler’s requirements of nutrients and energy according to the breeder’s recommendations [32]. For diet preparation, three different basal feed mixtures for the starter, grower and finisher period, respectively, were produced from all feed components except the fat source using mixing machines (V100 and V250, Diosna, Germany). The experimental diets were obtained by mixing the different basal feed mixtures with the intended amounts of fat sources (soybean oil, HI fat) using the mixing machine. Prior to adding the HI fat, the HI fat was melted in an electric oven at 50 °C. Subsequently, the experimental diets were pelleted using a pelleting device (V3/30 C, Simon-Heesen, Boxtel, Netherlands) and aliquots of all diets were stored at −20 °C for analysis of diet composition. Diets were fed in crumbled form during the first 3 d, and in pellet form (2 mm diameter) from d 3 until the end of the trial. Body weight (individually) and feed intake (per cage) were determined on d 1, 10, 21 and 35, and the feed:gain ratio was calculated from feed intake and body weight gains on cage basis.

**Table 1:** Composition of the broiler diets, g/kg

Item	Starter diets			Grower diets			Finisher diets		
	HI-0	HI-2.5	HI-5.0	HI-0	HI-2.5	HI-5.0	HI-0	HI-2.5	HI-5.0
Maize	300	300	300	280	280	280	280	280	280
Soybean meal (44% CP)	380	380	380	320	320	320	300	300	300
Wheat	205	205	205	283.5	283.5	283.5	308.65	308.65	308.65
Soybean oil	50	25	-	50	25	-	50	25	-
HI fat	-	25	50	-	25	50	-	25	50
Mineral & vitamin mix – Starter^*^	20	20	20	-	-	-	-	-	-
Mineral & vitamin mix – Grower^*^	-	-	-	20	20	20	-	-	-
Mineral & vitamin mix – Finisher^*^	-	-	-	-	-	-	20	20	20
Monocalciumphosphate	15	15	15	15	15	15	9	9	9
Calciumcarbonate	15.5	15.5	15.5	15.5	15.5	15.5	15	15	15
Sodiumchloride	4	4	4	4	4	4	4	4	4
*DL*-Methionine	3.2	3.2	3.2	3	3	3	2.6	2.6	2.6
*L*-Lysine	2.1	2.1	2.1	2.5	2.5	2.5	1.8	1.8	1.8
*L*-Threonine	1.1	1.1	1.1	0.9	0.9	0.9	0.4	0.4	0.4
*L*-Arginine	1.4	1.4	1.4	1.3	1.3	1.3	0.5	0.5	0.5
*L*-Valine	1.5	1.5	1.5	2.5	2.5	2.5	1.6	1.6	1.6
*L*-Isoleucine	1.2	1.2	1.2	1.8	1.8	1.8	1.45	1.45	1.45
TiO_2_	-	-	-	-	-	-	5	5	5

^*^The mineral & vitamin mix supplied the following minerals and vitamins per kg diet (starter/grower/finisher): Fe, 40/40/40 mg; Cu, 15/15/15 mg; Mn, 100/100/100 mg; Zn, 100/100/100 mg; I, 1/1/1 mg; Se, 0.35/0.35/0.35 mg; vitamin A, 10,000/10,000/10,000 IU; vitamin D_3_, 5000/5000/5000 IU; vitamin K_3_, 3/3/3 mg; vitamin E, 80/50/50 IU; vitamin B_1_, 3/2/2 mg; vitamin B_2_, 9/8/6 mg; vitamin B_6_, 4/3/3 mg; vitamin B_12_, 0.02/0.015/0.015 mg; biotin, 0.2/0.18/0.18 mg; folic acid, 2/2/1.5 mg; nicotinic acid, 60/50/50 mg; choline chloride, 500/400/350 mg; pantothenic acid, 15/12/10 mg

The experimental diets did not contain any technological (e.g., emulsifier) or zootechnical feed additives (e.g., feed enzymes)

### Analysis of diet composition

Concentrations of DM, crude protein, crude ash, ether extract, crude fiber and amino acids in the main diet components (wheat, maize, soybean extraction meal) and the experimental diets were determined by official methods [33]. Concentrations of sugar and starch were analyzed in the experimental diets by official methods (sugar according to method 7.2.1 and starch according to method 7.1.3 [33]). Total lipid fatty acid composition of HI fat, soybean oil and diets were analyzed as described below. Apart from total lipid fatty acid composition, the detailed composition (all major lipid classes and individual lipid species) of the HI fat was comprehensively analyzed using lipidomics, as described for liver and plasma (see below). The apparent N-corrected metabolizable energy (AME_N_) content of the diets was calculated according to the formula of the World’s Poultry Science Association (WPSA) for poultry compound feed [34]:

$${\mathrm{AME}}_{\mathrm N}\;(\mathrm{MJ}/\mathrm{kg})\:=\left[\left(0.01551\:\times\:\mathrm{crude}\;\mathrm{protein}\right)\:+\left(0.03431\:\times\:\mathrm{ether}\;\mathrm{extract}\right)\:+\;\left(0.01669\:\times\:\mathrm{starch}\right)\:+\:\left(0.01301\:\times\:\mathrm{sugar}\right)\right]$$

Considering that the AME and AME_N_ content of HI larvae fat for broiler chickens was shown to be similar to that of soybean oil [35], the formula is appropriate to calculate the ME contents of the experimental diets.

### Sample collection

A total of 12 (group HI-0) and 14 (groups HI-2.5 and HI-5.0) broilers per group (two broilers from each cage), whose body weights represented the mean body weight of the whole group, were selected for sample collection in order to avoid that effects were biased by random selection of broilers with very low or very high body weights. All analyses described below were carried out in these animals. The animals were killed by bleeding (opening of *Vena jugularis* and *Arteria carotis*) under electrical anesthesia using a BTG-40A stunning device (Westerhoff Geflügeltechnik, Hoogstede, Germany) in accordance with the European legislation for euthanasia of animals [36]. Whole blood was collected into ethylenediaminetetraacetic acid-coated polyethylene tubes (9 mL S-Monovette, Sarstedt, Nümbrecht, Germany). Plasma was prepared by centrifugation (1100 × *g*, 10 min) at 4 °C and stored at −20 °C. The liver was excised, washed in ice-cold NaCl solution (0.9%), weighted and small aliquots were snap-frozen in liquid nitrogen and stored at −80 °C. The gastrointestinal tract was removed and digesta from the ileum (segment between Meckel´s diverticulum and the ileo-cecal junction) and the cecum was collected. Tissue and digesta samples were snap-frozen in liquid nitrogen and stored at −80 °C pending analysis.

### Determination of AID coefficient for ether extract

The AID coefficient for ether extract was determined at the end of the experiment by the indicator method using titanium dioxide as an inert marker [31]. Prior to analysis, ileal digesta samples were freeze-dried and ground using a centrifugal mill (Retsch, Haan, Germany). Ileal digesta concentration of the indigestible indicator TiO_2_ was determined by the method of Brandt and Allam with slight modifications [37]. Concentration of ether extract in the ileal digesta was determined by official methods as described above. Based on the ileal concentrations of indicator, the AID coefficient for ether extract was calculated according to the following formula:

$$\mathrm{AID}\;\mathrm{coefficient}\;(\%)\:=\:100-\lbrack({\mathrm{TiO}}_{2_\_\mathrm{Diet}}/{{\mathrm{TiO}}_2}_{{}_\_\mathrm{Di}\mathrm g\mathrm e\mathrm s\mathrm t\mathrm a})\:\times\:({{\mathrm{EE}}_\_}_{\mathrm{Digesta}}/{\mathrm{EE}}_{{}_\_\mathrm{Di}\mathrm e\mathrm t})\:\times\:100\rbrack,$$

in which TiO_2_Diet_ is the TiO_2_ concentration in the diet (% DM), TiO_2_Digesta_ is the TiO_2_ concentration in the ileal digesta (% DM), EE__Digesta_ is the ether extract concentration in ileal digesta (% DM), and EE__Diet_ is the ether extract concentration in the diet (% DM).

### Determination of microbiota composition and diversity in the cecal digesta

Metagenomic DNA was isolated from approximately 180–200 mg of cecal digesta using genomic DNA columns (Macherey‐Nagel, Düren, Germany) according to Lagkouvardos et al. [38]. V3-V4 regions of the 16S rRNA genes were amplified using bacteria‐specific primers following a two-step procedure according to the Illumina sequencing protocol as described [38]. Amplicons were sequenced using a MiSeq system (Illumina, Inc., San Diego, CA, USA). Further processing of raw sequences was carried out as described recently [39]. Finally, sequences with a relative abundance > 0.1% in at least one sample were sorted, merged and operational taxonomic units (OTU) were picked at a threshold of 97% similarity. Taxonomic classification to the OTU was assigned using the SILVA database [40]. Further downstream analyses were done using Rhea (https://lagkouvardos.github.io/Rhea/). The differential abundance analysis of taxa was performed on the aggregated data at the different taxonomic levels as described [38]. For estimation of diversity within samples (α-diversity), the Shannon and Simpson indices, the most common indices to compare diversity, were calculated and transformed to the corresponding effective number of species according to Jost [41], because they are better suited at indicating the true diversity between samples and are minimally affected by the number of rare species. To measure the similarity between different microbial profiles, the β-diversity was determined by calculating generalized UniFrac distances with PERMANOVA statistical test as described previously [38]. Visualization of bacterial profiles among different groups was done by computation of non-metric multidimension distance scaling (NMDS) [42].

### Determination of SCFA concentrations in the cecal digesta

Cecal digesta SCFA concentrations were determined as described previously [43]. In brief, 50 mg aliquots of cecal digesta were mixed with 0.5 mL 5% o-phosphoric acid containing internal standard (0.15 mg/mL crotonic acid). Extraction was carried out by vortexing for 3 min and subsequent centrifugation at 21,100 × *g* at 4 °C for 10 min. Prior to injection, the supernatant was centrifuged again at 21,100 × *g* at 4 °C for 5 min. 1 μL of the extract was injected into a gas chromatograph (Clarus 580 GC system, Perkin Elmer, Waltham, USA) equipped with a polar capillary column (10 m free fatty acid phase, 0.32 mm internal diameter, 0.25 μm film thickness; Macherey and Nagel, Düren, Germany) and a flame ionisation detector.

### RNA extraction and hepatic transcript profiling

Total RNA from liver aliquots (20 mg) were isolated using TRIzol reagent (Invitrogen, Karlsruhe, Germany) according to the manufacturer’s protocol. RNA quantity and quality were assessed spectrophotometrically using an Infinite 200 M microplate reader equipped with a NanoQuant plate (both from Tecan, Mainz, Germany). The average RNA concentration and the A_260_/A_280_ ratio of all total RNA samples (*n* = 40, means ± SD) were 421 ± 48 ng/μL and 1.90 ± 0.02. For hepatic transcript profiling, total RNA samples from six randomly selected broilers/group were sent on dry-ice to the Genomics Core Facility “KFB—Center of Excellence for Fluorescent Bioanalytics” (Regensburg, Germany). Following a further RNA quality check using an Agilent 2100 Bioanalyzer (Agilent Technologies, Waldbronn, Germany), which revealed an average RNA integrity number (RIN) value of 8.23 ± 0.19 for all samples (*n* = 18, means ± SD), total RNA samples were processed using an Affymetrix GeneChip Array (Chicken Gene 1.0 ST), which covers 18,214 genes represented by 439,582 probes, according to the Applied Biosystems™ GeneChip™ Whole Transcript (WT) PLUS Reagent Kit User Guide (Thermo Fisher Scientific, Waltham, MA, USA). Following scanning of the processed GeneChips, cell intensity files, which provided a single intensity value for each probe cell, were generated from the image data using the Command Console software (Affymetrix). The compressed array image files (CEL files) were imported into the Applied Biosystems™ Transcriptome Analysis Console (TAC) (v. 4.0.2) software (Thermo Fisher Scientific) for calculation of summarized probe set signals (in log2 scale) using the Robust Multichip Analysis algorithm, comparison fold changes (FC) and significance *P*-values (ANOVA). Annotation of the microarrays was performed with the “ChiGene-1_0-st-v1.na36.galgal3.transcript.csv” annotation file. The microarray data of this study have been deposited in MIAME compliant format in the NCBI´s Gene Expression Omnibus public repository [44]. Owing to the rather moderate differences in the hepatic transcriptomes between groups HI-5.0 vs. HI-0 and groups HI-2.5 vs. HI-0, the differentially expressed transcripts were filtered based on a fold change > 1.5 or < −1.5 and a *P*-value < 0.05. Identical or similar filter criteria were also applied in several recent studies [45, 46]. Filtering of differentially expressed transcripts using the Benjamini & Hochberg false discovery rate adjustment method could not be applied, because the adjusted *P*-values for all transcripts were > 0.05.

Gene set enrichment analysis (GSEA) was performed with the identified differentially expressed transcripts in order to identify enriched Gene Ontology (GO) terms within GO category biological process using the Database for Annotation, Visualization and Integrated Discovery (DAVID) 6.8 bioinformatic resource [47, 48]. Biological process and molecular function terms were considered as enriched if *P* < 0.05.

### Validation of microarray data using qPCR analysis

Microarray data of 16 differentially expressed transcripts were validated by qPCR. For qPCR analysis, total RNA from all broilers (*n* = 12–14/group) was used. Synthesis of cDNA and qPCR analysis was performed with a Rotor-Gene Q system (Qiagen, Hilden, Germany) as described recently in detail [49]. Gene-specific primers were synthesized by Eurofins MWG Operon (Ebersberg, Germany). Characteristics of primers are listed in Additional file 1: Table S1. Normalization was carried out using multiple reference genes as described recently [50].

### Tissue homogenization and lipid extraction

Frozen liver tissue was homogenized in methanol/water (50/50, v/v) with addition of 1% sodium laurylsulfate using bead-based homogenization at a concentration of 0.05 mg/µL [51]. Lipid class specific, non-endogenous internal standards were added prior to lipid extraction. An amount of 2 mg liver (wet weight) or a volume of 10 µL plasma was subjected to lipid extraction according to the protocol by Bligh and Dyer [52]. A volume of 0.5 mL of the chloroform phase was recovered by a pipetting robot and vacuum dried. The residue was dissolved in 1.2 mL chloroform/methanol/2-propanol (1:2:4, v/v/v) with 7.5 mmol/L ammonium formate.

### Lipidomic analysis of major lipid classes by mass spectrometry

The analysis of lipids was performed by direct flow injection analysis (FIA) using a high-resolution Fourier Transform (FT) hybrid quadrupole-Orbitrap mass spectrometer (FIA-FTMS) [53]. TG, diglycerides (DG) and cholesteryl esters (CE) were recorded in positive ion mode as [M + NH_4_]^+^ in *m/z* range 500–1000 and a target resolution of 140,000 (at *m/z* 200). CE species were corrected for their species-specific response [54]. Ceramides (Cer), phosphatidylcholines (PC), ether PC (PC O), phosphatidylethanolamines (PE), ether PE (PE O), phosphatidylglycerols (PG), phosphatidylinositols (PI), and sphingomyelins (SM) were analyzed in negative ion mode in *m/z* range 520–960; lysophosphatidylcholines (LPC) and lysophosphatidylethanolamine (LPE) in *m/z* range 400–650. Multiplexed acquisition (MSX) was applied for free cholesterol (FC) and the internal standard FC[D7] [54]. Lipid annotation is based on the latest update of the shorthand notation [55].

The datasets from liver and plasma lipidomes were subjected to principal component analysis (PCA) using the MetaboAnalystR 3.2 package for R version 4.2.1. For the PCA, the relative metabolite composition of individual lipid species within the different lipid classes were used. Prior to the PCA, variables with missing values were either excluded from the analyzes if more than 50% of the samples were missing or the missing values were replaced by the limit of detection (1/5 of the minimum positive value of each variable). After normalization by log transformation and autoscaling the remaining values were used for the PCA.

### Determination of fatty acid composition of total lipids of the diets and the liver

Fatty acid composition of total lipids of the liver and the diets was determined by gas chromatography-flame ionization detection (GC-FID). Briefly, total lipids were extracted from 75 mg liver aliquots with a 3:2 (v/v)-mixture of n-hexane and isopropanol containing C19:0 (50 mg/mL) as internal standard. After extraction, samples were centrifuged (1200 × *g*, 10 min) and an aliquot of the supernatant was evaporated under a stream of N_2_ at 37 °C. Lipids were subsequently transmethylated using trimethylsulfonium hydroxide solution (Sigma-Aldrich) and the resulting fatty acid methyl esters (FAME) were separated by a GC-FID system described in detail recently [56].

### Statistical analysis

Statistical analysis was conducted with SPSS 27 software (IBM, Armonk, NY, USA). The cage served as the experimental unit for feed intake and feed:gain ratio and the individual animal for all other data. All parameters were tested with the Shapiro–Wilk test for normal distribution and with the Levene’s test for homoscedasticity. When the normal distribution was followed only after a log transformation, the log transformed data were used for statistical analysis. Differences between the three groups were analyzed by one-way analysis of variance (one-way ANOVA) followed by a Tukey’s post-hoc test when the data were normally distributed and the variances were homogeneous. If the data showed heterogeneity of variance, the means of the three groups were analyzed using Welch's ANOVA in conjunction with the post-hoc Games-Howell test. If the normal distribution was not followed, a Kruskal–Wallis one-way ANOVA was performed using the Mann–Whitney U test with Bonferroni correction as post-hoc test. For all tests, a* P*-value < 0.05 was considered statistically significant.

## Results

### Lipid composition of the HI fat

Analysis of the composition of major lipid classes of the HI fat revealed that HI total lipids consisted almost completely of TG (99.2% of total fat). All other lipid classes detected (PG, PC, DG, PE and SM) made up < 0.3% of total lipids (Table 2). Analysis of individual TG species demonstrated that TG 36:0, TG 38:0, TG 40:0 and TG 42:2 were the most abundant TG species, whereas all other TG species contributed < 5% of all TG species (Table 2). The majority of TG species of the HI fat was saturated (56.7%), while TG species with one, two and three or more double bonds made up 14.0%, 17.3% and 12.0%, respectively. Due to the low proportions of PG, PC, DG, PE, PC-O, SM and LPC in the HI fat, the individual lipid composition of these non-TG lipid classes is not reported. Analysis of the fatty acid composition of HI fat by GC-FID revealed that SFA were the dominating fatty acids, with C12:0 (57.2%), C14:0 (8.7%) and C16:0 (10.9%) contributing to 76.8% of total fatty acids (data not shown). The essential fatty acids C18:2 n-6 and C18:3 n-3 made up 10.6% and 0.8%, of total fatty acids, respectively.

**Table 2:** Lipid composition of the *Hermetia illucens* larvae fat

Item	Content
Lipid class, % of total lipids	
TG	99.20 ± 0.02
PG	0.28 ± 0.01
PC	0.18 ± 0.01
DG	0.17 ± 0.01
PE	0.12 ± 0.02
SM	0.02 ± 0.01
TG species, % of total TG species^a^	
34:0	2.06 ± 0.04
36:0	30.06 ± 0.49
37:0	0.60 ± 0.31
38:0	12.25 ± 0.51
38:1	0.59 ± 0.01
40:0	7.76 ± 0.39
40:1	2.40 ± 0.10
42:0	2.11 ± 0.22
42:1	3.91 ± 0.08
42:2	6.04 ± 1.04
42:3	0.73 ± 0.07
44:0	0.92 ± 0.10
44:1	2.10 ± 0.21
44:2	1.65 ± 0.09
46:1	2.54 ± 0.43
46:2	4.80 ± 0.21
46:3	0.95 ± 0.03
48:1	0.92 ± 0.21
48:2	1.79 ± 0.15
48:3	1.78 ± 0.07
48:4	1.89 ± 0.18
50:2	1.21 ± 0.06
50:3	0.86 ± 0.03
52:2	0.56 ± 0.09
52:3	1.13 ± 0.02
52:4	1.30 ± 0.17
Sum DB0	56.73 ± 1.16
Sum DB1	13.99 ± 0.98
Sum DB2	17.29 ± 1.26
Sum DB3	6.33 ± 0.16
Sum DB4	4.18 ± 0.39
Sum DB5	0.94 ± 0.11
Sum DB6	0.45 ± 0.08
Sum DB7	0.09 ± 0.01

Abbreviations: *DB* Double bond, *DG* Diglycerides, *LPC *Lysophosphatidylcholine, *PC *Phosphatidylcholine, *PC O* PC-ether, *PE* Phosphatidylethanolamine, *PG* Phosphatidylglycerol, *SM* Sphingomyelin, *TG* Triglycerides

^a^Only TG species > 0.5% are shown. Data are means ± SD, *n* = 4 samples

### Composition of the experimental diets

The three experimental diets within each feeding phase had similar concentrations of crude nutrients, sugar, starch and energy (Table 3) and amino acids (Additional file 1: Table S2), but substantially differed in the fatty acid composition of dietary total lipids (Table 3). With increasing replacement of soybean oil by HI fat, the proportions of C10:0, C12:0 and C14:0 markedly increased, while those of C18:0, C18:1, C18:2 n-6 and C18:3 n-3 decreased. As a consequence, the dominating fatty acids in the HI-5.0 diet were in decreasing order: C12:0, C18:2 n-6, C18:1, C16:0 and C14:0; in the HI-2.5 diet the dominating fatty acids were in decreasing order: C18:2 n-6, C18:1, C12:0, C16:0 and C18:3 n-3; in the HI-0 diet the dominating fatty acids were in decreasing order: C18:2 n-6, C18:1, C16:0, C18:3 n-3 and C18:0. Monounsaturated fatty acids and polyunsaturated fatty acids in the HI fat total lipids made up 9.4% and 11.4%, respectively.

**Table 3:** Concentrations of nutrients and energy in the broiler diets

Item	Starter diets			Grower diets			Finisher diets		
	HI-0	HI-2.5	HI-5.0	HI-0	HI-2.5	HI-5.0	HI-0	HI-2.5	HI-5.0
Analyzed crude nutrient content									
Dry matter, % FM	87.5	87.5	87.4	87.6	87.3	87.7	87.5	87.4	88.1
Crude protein, % DM	21.6	21.6	21.6	19.8	20.0	20.0	19.1	19.0	19.3
Ether extract, % DM	7.3	7.1	7.2	7.6	7.4	7.4	7.6	7.4	7.2
Crude ash, % DM	5.6	5.9	5.9	5.5	5.5	5.6	5.5	5.4	5.5
Crude fiber, % DM	3.5	3.6	3.5	3.9	3.9	3.9	4.2	4.3	4.0
Sugar, % DM	3.3	3.0	2.7	3.8	3.0	2.7	2.9	3.0	3.4
Starch, % DM	35.7	35.9	35.5	37.2	36.4	37.7	36.4	38.6	38.3
AME_N_^#^, MJ/kg DM	12.2	12.2	12.1	12.4	12.1	12.3	12.0	12.3	12.3
Fatty acids^a^, % of total fatty acids									
C10:0	-	0.41	0.82	-	0.46	0.81	-	0.44	0.80
C12:0	0.42	18.23	37.65	0.22	19.06	38.09	0.50	19.06	38.03
C14:0	0.18	2.93	5.89	0.15	3.09	5.97	0.20	3.08	6.05
C16:0	11.82	11.88	12.17	11.44	12.06	12.19	11.59	12.12	12.26
C16:1	0.22	1.00	1.90	0.16	1.02	1.92	0.16	1.07	1.92
C18:0	3.99	3.10	1.85	3.98	3.09	1.86	3.89	3.09	1.87
C18:1	24.00	19.44	12.42	25.57	18.65	12.60	25.00	19.00	12.56
C18:2 n-6	51.32	37.73	22.94	50.88	36.83	23.13	50.93	37.26	23.02
C18:3 n-3	5.66	3.74	1.65	5.48	3.55	1.64	5.55	3.56	1.62
C20:0	0.51	0.38	0.28	0.46	0.37	0.28	0.45	0.34	0.28

*Abbreviations: AME*_*N*_ Apparent N-corrected metabolizable energy, *DM *Dry matter, *FM *Fresh matter

^a^Only fatty acids > 0.5% of total fatty acids are shown. ^#^The AME_N_ content of the feed was calculated according to the formula of the World´s Poultry Science Association for poultry compound feed

### Performance and ether extract digestibility

Performance data (body weight gain, final body weight, feed intake, and feed:gain ratio) during the whole period were not different between groups. However, body weight gain and feed intake during the starter period were higher in groups HI-2.5 and HI-5.0 than in group HI-0 (*P* < 0.05, Table 4). No differences of performance data between groups were found during the grower and the finisher period. AID coefficient for ether extract also did not differ between groups.

**Table 4:** Performance data and apparent ileal digestibility (AID) coefficient for ether extract of broilers fed diets with either 0 (HI-0), 2.5% (HI-2.5) or 5.0% (HI-5.0) *Hermetia illucens* (HI) larvae fat for 35 d

Item	HI-0	HI-2.5	HI-5.0	*P*-value
Whole period (d 1 to 35)				
Initial BW, g	44.8 ± 0.4	44.7 ± 0.9	45.1 ± 0.9	0.719
Final BW, g	2740 ± 139	2696 ± 237	2738 ± 92	0.860
BW gain, g	2696 ± 138	2651 ± 237	2693 ± 92	0.861
Feed intake, g	3745 ± 183	3696 ± 236	3719 ± 127	0.898
Feed:Gain ratio, g/g	1.39 ± 0.02	1.40 ± 0.11	1.38 ± 0.03	0.537
Starter period (d 1 to 10)				
BW gain, g	272 ± 17^b^	290 ± 10^a^	287 ± 12^a^	0.049
Feed intake, g	290 ± 19^b^	307 ± 8^a^	308 ± 11^a^	0.042
Feed: Gain ratio, g/g	1.09 ± 0.02	1.09 ± 0.01	1.08 ± 0.01	0.437
Grower period (d 11 to 21)				
BW gain, g	823 ± 37	830 ± 61	819 ± 34	0.901
Feed intake, g	1068 ± 53	1081 ± 61	1065 ± 43	0.842
Feed: Gain ratio, g/g	1.30 ± 0.01	1.30 ± 0.04	1.30 ± 0.03	0.926
Finisher period (d 22 to 35)				
BW gain, g	1601 ± 91	1522 ± 210	1587 ± 79	0.560
Feed intake, g	2387 ± 124	2309 ± 181	2346 ± 98	0.612
Feed: Gain ratio, g/g	1.49 ± 0.03	1.54 ± 0.23	1.48 ± 0.06	0.822
AID coefficient for EE, %	89.0 ± 1.6	88.6 ± 1.5	88.8 ± 2.8	0.950

Data are means ± SD, *n* = 6–7 cages/groups. Abbreviation: *BW *Body weight, *EE *Ether extract

### Cecal microbiota diversity and composition

**Fig. 1:**
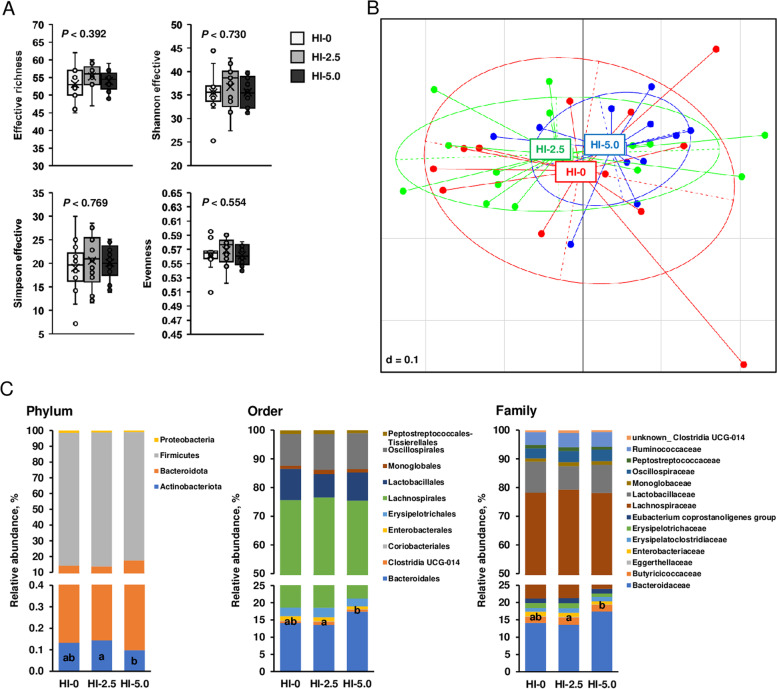
Analysis of the cecal microbiome. Indicators of α-diversity (effective richness, shannon effective, simpson effective, evenness) of the cecal bacterial community (**A**), visualization of the difference in β-diversity of cecal bacterial community between groups by non-metric multidimension distance scaling plot (**B**), and distribution of cecal bacteria at different taxonomic levels (phylum, order, family) (**C**) of broilers fed diets with either 0 (HI-0), 2.5% (HI-2.5) or 5.0% (HI-5.0) *Hermetia illucens* (HI) larvae fat for 35 d. **A**: Box and whisker plots for *n* = 12–14 broilers/group; **C**: Data are means for *n* = 12–14 broilers/group. ^a,b^Means without a common letter differ across the groups, *P* < 0.05

In order to identify alterations of the cecal microbiota structure of the broilers, 16S rRNA-based high-throughput sequencing was applied. Following quality check, chimera check and filtering, the high-quality sequences obtained from the cecum digesta samples of the 40 broilers were delineated into 90 OTUs at 97% sequence identity (Additional file 1: Table S3). Treatment effect on microbial diversity was evaluated by the use of different diversity metrics. None of the metrics used to describe α-diversity (effective species richness, shannon effective, simpson effective, evenness) differed between groups (Fig. 1a). In addition, β-diversity of cecal bacterial community calculated based on generalized UniFrac distances did not differ among groups. The MetaNMDS plot generated to visualize the difference in β-diversity of cecal bacterial community among groups shows that no clustering was most visible among groups (Fig. 1b).

To analyze the effect on the microbiota composition, the microbial community was analyzed at different taxonomic levels (phylum, class, order, family, genus). In total, only the relative abundances of four different bacterial taxa differed among groups. At the phylum level, only the relative abundance of the least abundant bacterial phylum, Actinobacteriota, contributing less than 0.15% to total bacterial phyla was different among groups. While the relative abundance of this phylum was lower in group HI-5.0 than in group HI-2.5 (*P* < 0.05), it did not differ between group HI-5.0 and HI-0 and between group HI-2.5 and HI-0 (Fig. 1c). Amongst five different bacterial classes identified in the cecal digesta of the broilers, only the relative abundance of Coriobacteriia, which belong to the phylum Actinobacteriota, was affected by treatment; while the relative abundance of this phylum was lower in group HI-5.0 than in group HI-2.5 (*P* < 0.05), it did not differ between group HI-5.0 and HI-0 and between group HI-2.5 and HI-0. At the order level, the relative abundance of Coriobacteriales (class Coriobacteriia) was identified as the only bacterial order differing between groups; its relative abundance was lower in group HI-5.0 than in group HI-2.5 (*P* < 0.05), but was not different between groups HI-5.0 and HI-2.5 (Fig. 1c). At the family level, the only taxon identified to be altered was the Eggerthellaceae (order Coriobacteriales), whose abundance was lower in group HI-5.0 than in group HI-2.5 (*P* < 0.05), but was not different between group HI-5.0 and HI-0 and between group HI-2.5 and HI-0 (Fig. 1c). No differences between groups were found regarding the relative abundances of bacterial genera.

### Cecal concentrations of short-chain fatty acids

**Fig. 2:**
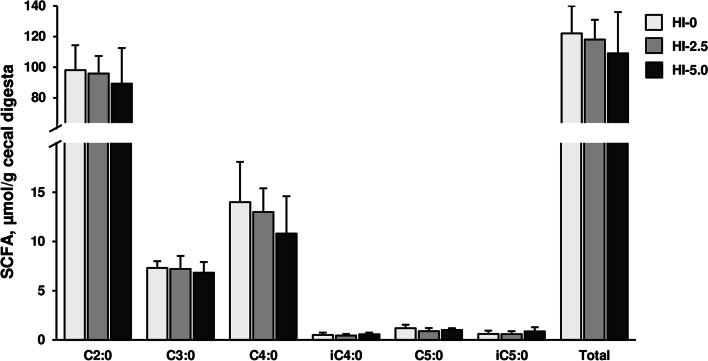
Analysis of microbial fermentation profile in the gut. Concentrations of individual [acetic acid (C2:0), propionic acid (C3:0), butyric acid (C4:0), isobutyric acid (iC4:0), valeric acid (C5:0), isovaleric acid (iC5:0)] and total (= sum of individual) short-chain fatty acids (SCFA) in the cecal digesta of broilers fed diets with either 0 (HI-0), 2.5% (HI-2.5) or 5.0% (HI-5.0) *Hermetia illucens* (HI) larvae fat for 35 d. Data are means ± SD, *n* = 12–14 broilers/group

The concentrations of total and individual SCFA [acetic acid (C2:0), propionic acid (C3:0), isobutyric acid (iC4:0), butyric acid (C4:0), isovaleric acid (iC5:0) and valeric acid (C5:0)] in the cecal digesta were not different between the three groups (Fig. 2).

### Liver transcriptome

**Fig. 3:**
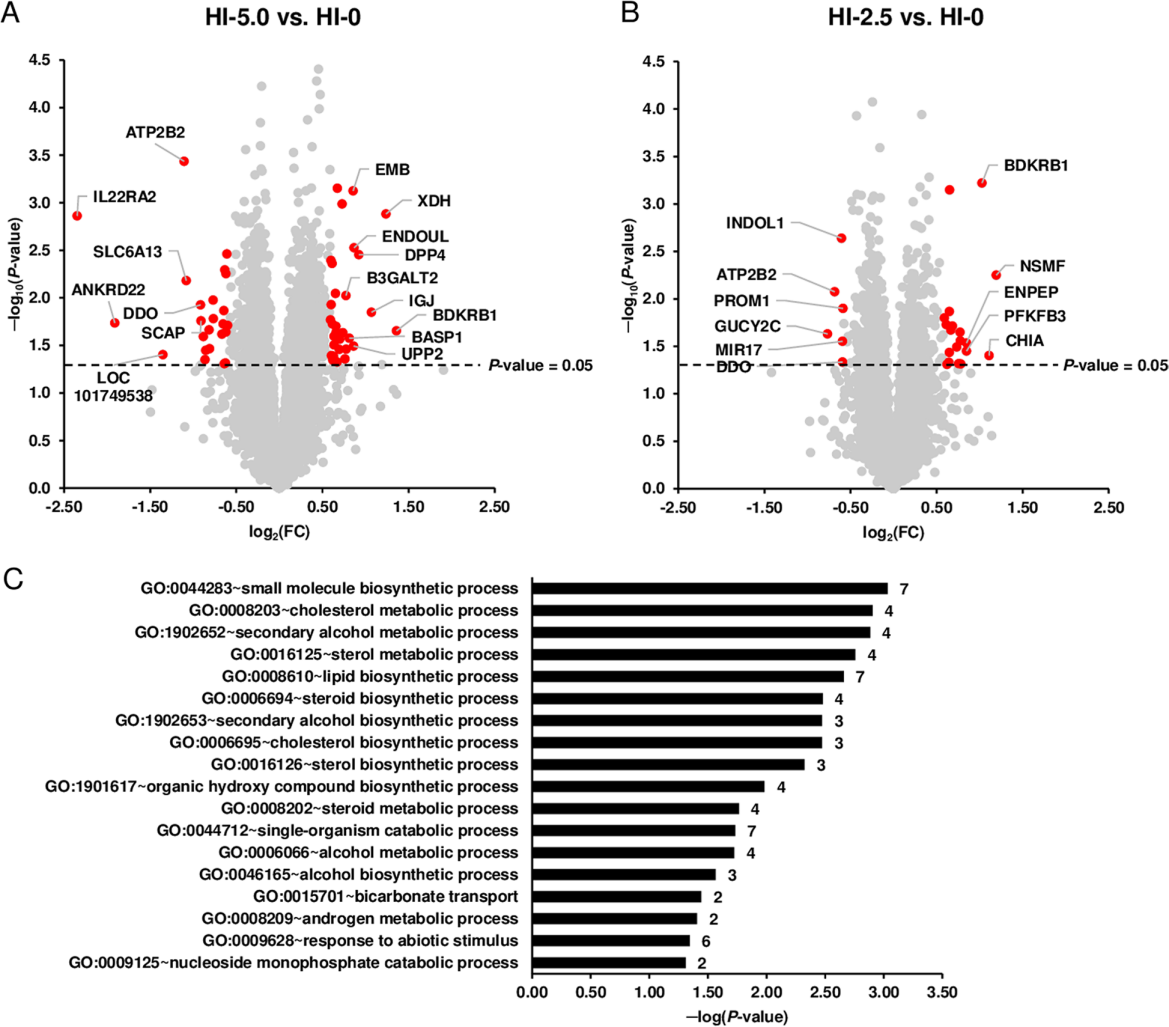
Differential transcriptome analysis of the liver. Volcano plot illustrating the differentially expressed transcripts in the liver of broilers between group HI-5.0 vs. HI-0 (**A**) and group HI-2.5 vs. HI-0 (**B**). The double filtering criteria are indicated by horizontal (*P*-value < 0.05) and vertical (fold change > 1.5 or < −1.5) dashed lines. Red dots in the upper left and the upper right corner represent the downregulated and the upregulated transcripts, respectively. **C** Most enriched gene ontology (GO) biological process terms associated with the transcripts differentially expressed between group HI-5.0 vs. HI-0. GO terms are sorted by their enrichment *P*-values (EASE score) (top: lowest *P*-value, bottom: highest *P*-value)

According to the filter criteria applied (*P* < 0.05; fold change > 1.5 and < −1.5), a total of 55 transcripts were identified as differentially expressed (upregulated: 31, downregulated: 24) in the liver between groups HI-5.0 and HI-0. In Fig. 3a, the differentially expressed transcripts between groups HI-5.0 and HI-0 are illustrated as red dots in the Volcano plot. Amongst the upregulated genes, only three genes (*BDKRB1*, *XDH*, *IGJ*) exhibited a regulation > 2.0-fold. The top 10 upregulated transcripts were in decreasing order of their fold change (in brackets): *BDKRB1* (2.57), *XDH* (2.36), *IGJ* (2.10), *DPP4* (1.90), *ENDOUL* (1.83), *UPP2* (1.82), *EMB* (1.81), *BASP1* (1.76), *SIK1* (1.71) and *B3GALT2*. Amongst the downregulated genes, five genes (*IL22RA2*, *ANKRD22*, *LOC101749538*, *ATP2B2*, *SLC6A13*) were regulated < −2.0-fold. The top 10 downregulated transcripts were in increasing order of their fold change: *IL22RA2* (−5.09), *ANKRD22* (−3.76), *LOC101749538* (−2.55), *ATP2B2* (−2.15), *SLC6A13* (−2.12), *DDO* (−1.89), *SCAP* (−1.88), *SPATA4* (−1.84), *CA4* (−1.82) and *ULK1* (−1.81). The fold change and *P*-value of all differentially expressed transcripts between group HI-5.0 vs. HI-0 are shown in Additional file 1: Table S4.

Considering the same filter criteria as for the comparison of groups HI-5.0 vs. HI-0, a total of 25 transcripts were identified as differentially expressed (upregulated: 19, downregulated: 6) in the liver between groups HI-2.5 and HI-0 (Fig. 3b). Only three genes (*NSMF*, *CHIA*, *BDKRB1*) were regulated > 2.0-fold and no genes were regulated < −2.0-fold. The 10 most strongly upregulated transcripts were in decreasing order of their fold change (in brackets): *NSMF* (2.29), *CHIA* (2.16), *BDKRB1* (2.04), *ENPEP* (1.80), *PFKFB3* (1.80), *MIR1790* (1.72), *SIK1* (1.71), *DPP4* (1.71), *LOC395159* (1.69) and *CP* (1.66). The 6 downregulated transcripts were in increasing order of their fold change: *GUCY2C* (−1.53), *ATP2B2* (−1.53), *INDOL1* (−1.51), *MIR17* (−1.51), *DDO* (−1.51) and *PROM1* (−1.50). The fold change and *P*-value of all differentially expressed transcripts between groups HI-2.5 vs. HI-0 are shown in Additional file 1: Table S5.

Microarray data of 16 differentially expressed transcripts between groups HI-5.0 and HI-0 were validated by qPCR. As shown in Additional file 1: Table S6, the effect direction (positive or negative fold change) was the same between microarray and qPCR for all validated transcripts, whereas the effect size (value of fold change) differed to some extent for the validated transcripts between microarray and qPCR. Statistical analysis of qPCR data revealed that twelve transcripts were regulated either significantly (*CA4*, *DDO*, *IL22RA2*, *HMGCS1*, *SCAP*, *SH2D4A*, *ULK1*, *XDH*) or at a *P*-value < 0.1 (*AKR1D1*, *ANKRD22*, *MTHFS*, *OGN*), whereas four transcripts (*DPP4*, *FANCL*, *SIK1*, *SPATA4*) were not regulated.

In order to extract biological meaning from the transcripts differentially expressed between groups HI-5.0 and HI-0, GSEA was performed using GO category biological process. Due to the low number of differentially expressed transcripts, GSEA was not conducted for the comparison of groups HI-2.5 and HI-0. Within GO category biological process, the most enriched biological process terms assigned to the transcripts regulated between groups HI-5.0 and HI-0 were (in increasing order of their *P*-values): small molecule biosynthetic process, cholesterol metabolic process, secondary alcohol metabolic process, sterol metabolic process, lipid biosynthetic process, steroid biosynthetic process, cholesterol biosynthetic process and secondary alcohol biosynthetic process (Fig. 3c).

### Liver and plasma lipidomes

**Fig. 4:**
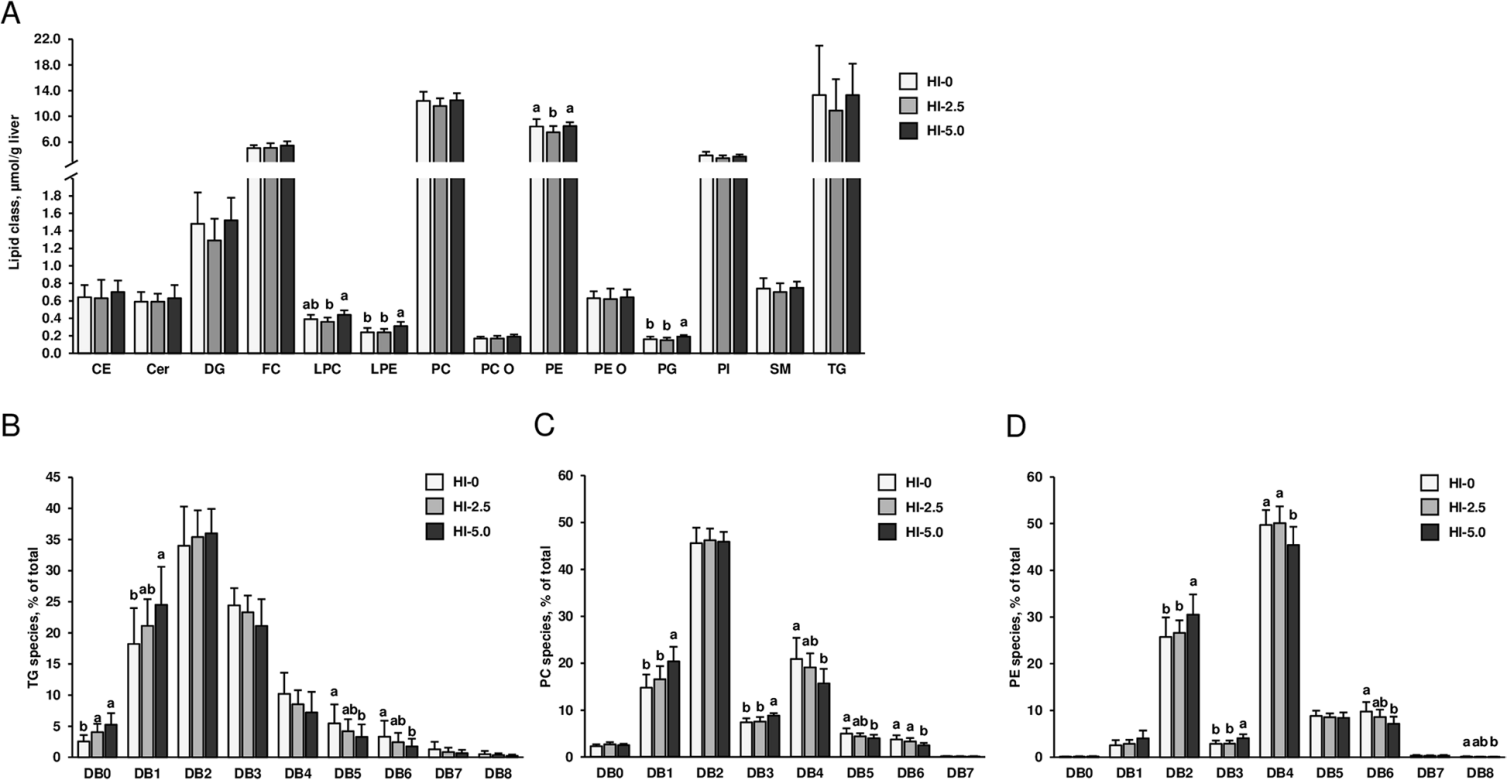
Analysis of the liver lipidome. Concentrations of the major lipid classes in the liver (**A**) and lipid species composition within the three most abundant lipid classes in the liver, TG (**B**), PC (**C**) and PE (**D**), of broilers fed diets with either 0 (HI-0), 2.5% (HI-2.5) or 5.0% (HI-5.0) *Hermetia illucens* (HI) larvae fat for 35 d. Lipid species were grouped according to the number of double bonds (DB). Data are means ± SD, *n* = 12–14 broilers/group. Abbreviations: CE, cholesteryl ester; Cer, ceramide; DG, diglycerides; FC, free cholesterol; LPC, lysophosphatidylcholine; LPE, lysophosphatidylethanolamine; PC, phosphatidylcholine; PC O, PC-ether; PE, phosphatidylethanolamine; PE O, PE-ether; PG, phosphatidylglycerol; PI, phosphatidylinositol; SM, sphingomyelin; TG, triglycerides

The major lipid classes in the liver were in decreasing order: TG, PC, PE, FC, PI, DG, SM, CE, PE O, Cer, LPC, LPE, PC O and PG. Despite that the sum of total lipid classes in the liver were not different between groups (HI-0: 47.6 ± 8.9 µmol/g, HI-2.5: 42.4 ± 8.9 µmol/g, HI-5.0: 47.7 ± 6.5 µmol/g, mean + SD, *n *= 12–14/group, *P *= 0.170), hepatic concentrations of PE, LPC, LPE and PG differed between groups (*P* < 0.05), even though only slightly (Fig. 4a). Hepatic concentrations of LPE and PG were higher in group HI-5.0 than in groups HI-0 and HI-2.5 (*P* < 0.05). Concentrations of PE and LPC in the liver were higher in group HI-5.0 than in group HI-2.5 (*P* < 0.05), but not compared to group HI-0.

In contrast to the slight or absent differences in the concentrations of the lipid classes in the liver between groups, pronounced differences between groups were seen with regard to the composition of individual lipid species within the different lipid classes. Regarding TG, the dominating lipid class in the liver, relative proportions of species with zero and one double bonds were higher in group HI-5.0 than in group HI-0, while those with five and six double bonds were lower in group HI-5.0 than in group HI-0 (*P* < 0.05, Fig. 4b). With regard to PC, the second most abundant hepatic lipid class, relative proportions of species with one and three double bonds were higher and those with four, five and six double bonds were lower in group HI-5.0 than in group HI-0 (*P* < 0.05, Fig. 4c). Within PE, the third most abundant lipid class in the liver, relative proportions of species with two and three double bonds were higher and those with four, six and eight double bonds were lower in group HI-5.0 than in group HI-0 (*P *< 0.05, Fig. 4d). The proportions of all individual TG, PC and PE species are shown in Additional file 1: Table S7.

**Fig. 5:**
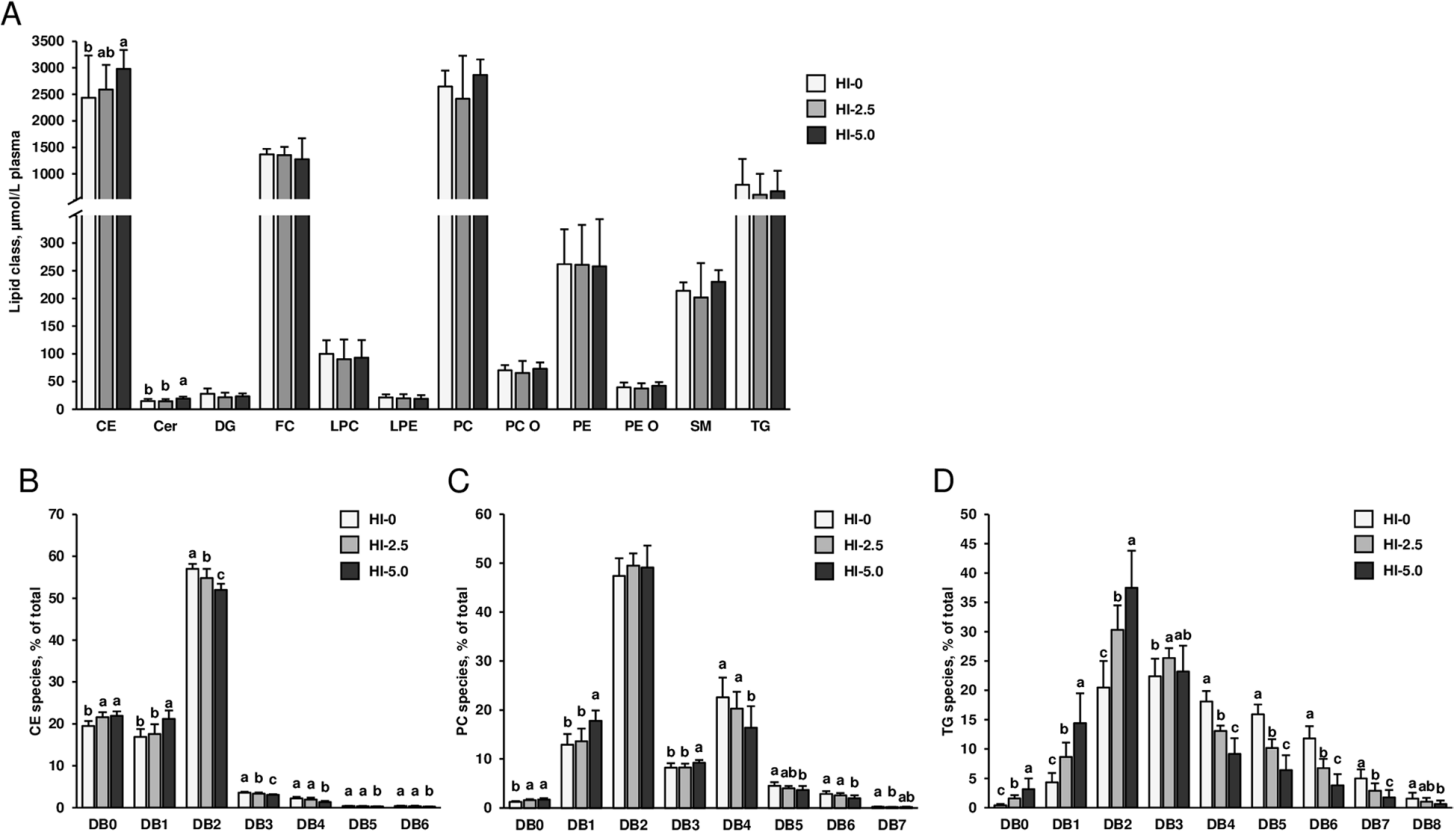
Analysis of the plasma lipidome. Concentrations of the major lipid classes in the plasma (**A**) and lipid species composition within the three most abundant lipid classes in plasma, CE (**B**), PC (**C**) and TG (**D**), of broilers fed diets with either 0 (HI-0), 2.5% (HI-2.5) or 5.0% (HI-5.0) *Hermetia illucens* (HI) larvae fat for 35 d. Lipid species were grouped according to the number of double bonds (DB). Data are means ± SD, *n* = 12–14 broilers/group. Abbreviations: CE, cholesteryl ester; Cer, ceramide; DG, diglycerides; FC, free cholesterol; LPC, lysophosphatidylcholine; LPE, lysophosphatidylethanolamine; PC, phosphatidylcholine; PC O, PC-ether; PE, phosphatidylethanolamine; PE O, PE-ether; SM, sphingomyelin; TG, triglycerides

In plasma, the major lipid classes were in decreasing order: PC, CE, FC, TG, PE, SM, LPC, PC O, PE O, DG, LPE and Cer (Fig. 5a). Like the concentrations of most lipid classes (PC, FC, TG, PE, SM, LPC, PC O, PE O, DG, LPE), the sum of total lipid classes in plasma did not differ among groups (HI-0: 7992 ± 1423 µmol/L, HI-2.5: 7683 ± 1742 µmol/L, HI-5.0: 8550 ± 752 µmol/L, mean + SD, *n* = 12–14/group, *P* = 0.358). In contrast, the plasma concentrations of CE and Cer differed among groups (*P* < 0.05), but only slightly; plasma concentrations of CE and Cer were higher in group HI-5.0 than in group HI-0. Plasma concentration of Cer in group HI-5.0 was also higher than in group HI-2.5 (*P* < 0.05), whereas plasma concentration of CE did not differ between group HI-5.0 and group HI-2.5. Similar as in the liver, pronounced differences among groups were observed regarding the composition of individual lipid species within the different lipid classes in plasma. With regard to CE, the dominating lipid class in the plasma, relative proportions of species with zero and one double bonds were higher in group HI-5.0 than in group HI-0, while those of species with two, three, four, five and six double bonds were lower in group HI-5.0 than in group HI-0 (*P* < 0.05, Fig. 5b). Regarding PC, the second most abundant plasma lipid class, the relative proportions of PC species with zero, one and three double bonds were higher, whereas those with four, five, six and seven double bonds were lower in group HI-5.0 than in group HI-0 (*P* < 0.05, Fig. 5c). With regard to TG, relative proportions of species with zero, one, and two double bonds were higher and those with four, five, six, seven and eight double bonds were lower in group HI-5.0 than in group HI-0 (*P* < 0.05, Fig. 5d). The proportions of all individual CE, PC and TG species in plasma are shown in Additional file 1: Table S8.

**Fig. 6:**
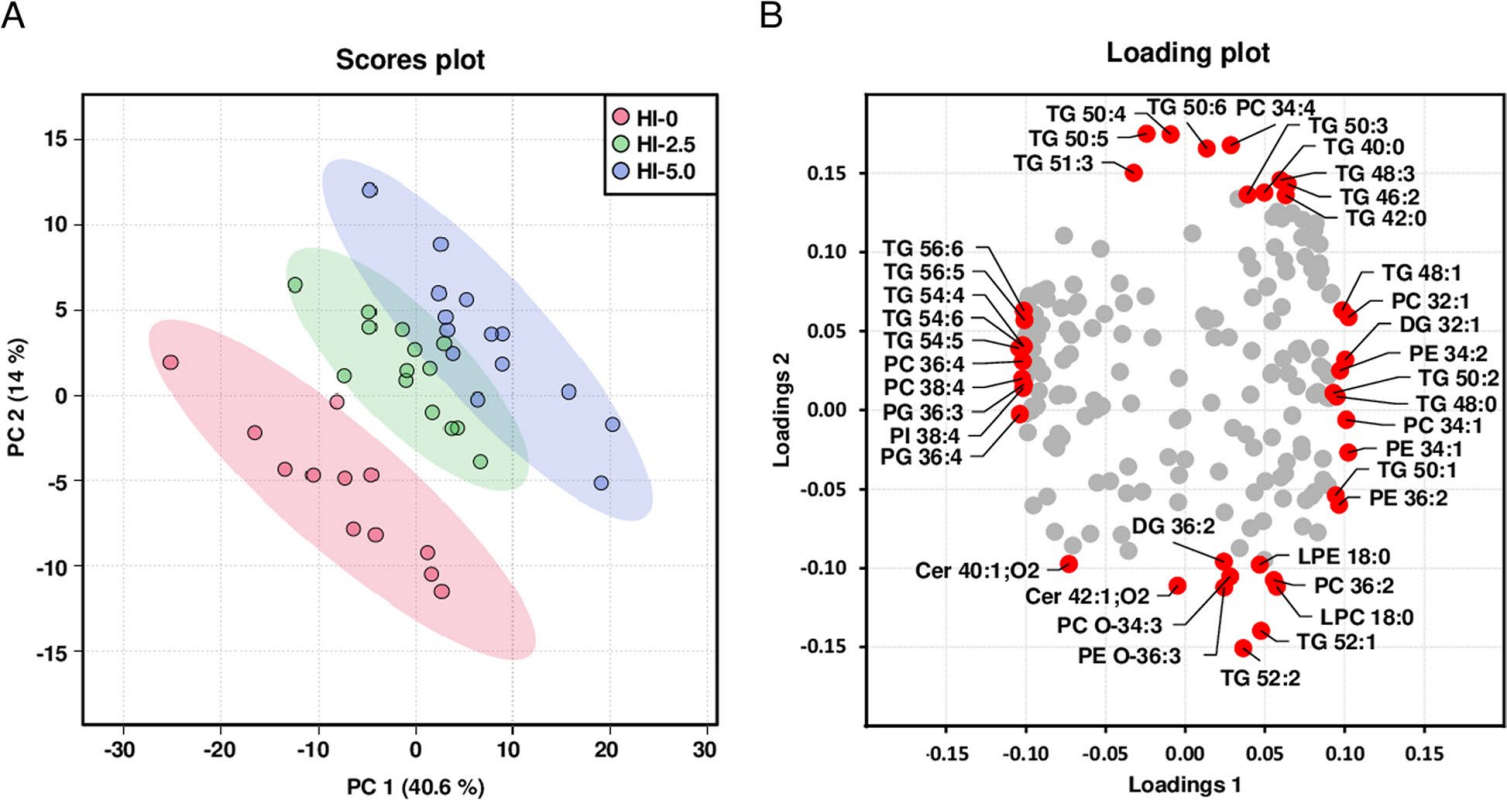
Principal component analysis of liver lipidome. Scores plot with plotted 5% confidence interval (**A**) and associated loading plot (**B**) of principal component analysis (PCA) of the liver lipidome of the broilers. Broilers were fed diets with either 0 (HI-0), 2.5% (HI-2.5) or 5.0% (HI-5.0) *Hermetia illucens* (HI) larvae fat for 35 d. Data are principal components (PC 1 or PC 2) and their loadings calculated based on the relative abundances of the lipid species within their lipid classes, *n* = 12–14 broilers/group. Abbreviations: Cer, ceramide; DG, diglycerides; LPC, lysophosphatidylcholine; LPE, lysophosphatidylethanolamine; PC, phosphatidylcholine; PC O, PC-ether; PE, phosphatidylethanolamine; PE O, PE-ether; PG, phosphatidylglycerol; PI, phosphatidylinositol; TG, triglycerides

**Fig. 7:**
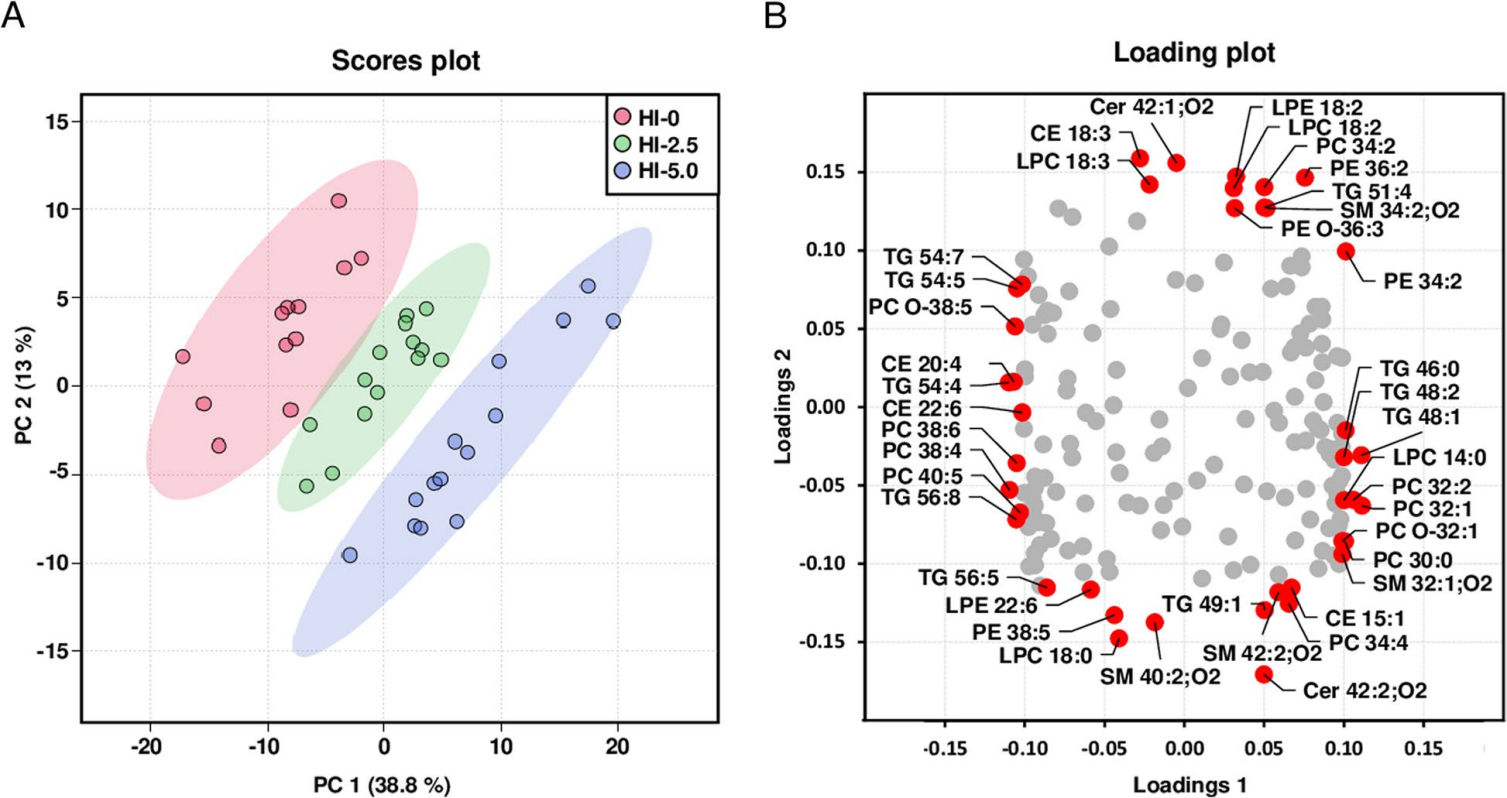
Principal component analysis of plasma lipidome. Scores plot with plotted 5% confidence interval (**A**) and associated loading plot (**B**) of principal component analysis (PCA) of the plasma lipidome of the broilers. Broilers were fed diets with either 0 (HI-0), 2.5% (HI-2.5) or 5.0% (HI-5.0) *Hermetia illucens* (HI) larvae fat for 35 d. Data are principal components (PC 1 or PC 2) and their loadings calculated based on the relative abundances of the lipid species within their lipid classes, *n* = 12–14 broilers/group. Abbreviations: CE, cholesteryl ester; Cer, ceramide; LPC, lysophosphatidylcholine; LPE, lysophosphatidylethanolamine; PC, phosphatidylcholine; PC O, PC-ether; PE, phosphatidylethanolamine; PE O, PE-ether; SM, sphingomyelin; TG, triglycerides

The pronounced differences among groups with regard to the composition of individual lipid species within the different lipid classes in liver and plasma were reflected by the results from PCA analysis which was carried out with the relative proportions of individual lipid species of all lipid classes. The dimensional reduction of the lipidome datasets from the liver (Fig. 6a) and the plasma (Fig. 7a) revealed a clear separation (rightward shift) between the three feeding groups with increasing dietary levels of HI fat. For the liver lipidome the cumulative proportion is 54.6% with principal component 1 accounting for more than 40% of the variance of the dataset. In the plasma, the cumulative proportion adds up to 51.8% with principal component 1 accounting for 38.8%. The loading plot for the liver lipidome dataset shows that the left shift of the HI-0 group was mainly caused by individual TG and PC lipid species with three or more double bonds and the right shift of the HI-2.5 and HI-5.0 groups was mainly caused by individual TG, PC and PE lipid species with less than 3 double bounds (Fig. 6b). In the plasma, the right shift of the HI-2.5 and HI-5.0 groups was largely driven by individual TG, PC and PE species with less than three double bounds, while the left shift of the HI-0 group is driven by individual TG, PC and CE species with more than 3 double bounds (Fig. 7b).

### Fatty acid composition of liver total lipids

As expected, fatty acid composition of hepatic total lipids was affected by dietary inclusion of HI fat. Proportions of C12:0, C14:0 and C16:1 were higher in group HI-5.0 than in groups HI-2.5 and HI-0, whereas proportion of C18:3 n-3 was lower in groups HI-5.0 and HI-2.5 than in group HI-0 (*P* < 0.05, Table 5).

**Table 5:** Fatty acid composition of hepatic total lipids of broilers fed diets with either 0 (HI-0), 2.5% (HI-2.5) or 5.0% (HI-5.0) *Hermetia illucens* (HI) larvae fat for 35 d

Fatty acid^*^, g/100 g total fatty acids	HI-0	HI-2.5	HI-5.0	*P*-value
C12:0	0.56 ± 0.17^c^	1.03 ± 0.2^b^	1.53 ± 0.34^a^	0.000
C14:0	0.48 ± 0.18^c^	1.18 ± 0.41^b^	1.89 ± 0.53^a^	0.000
C16:0	22.0 ± 2.7	22.4 ± 2.1	22.3 ± 2.4	0.945
C16:1	1.93 ± 0.68^b^	1.81 ± 0.85^b^	2.62 ± 0.55^a^	0.012
C18:0	21.5 ± 2.9	21.5 ± 2.4	19.7 ± 2.2	0.108
C18:1 n-9	21.6 ± 4.4	20.4 ± 4	23.1 ± 3.3	0.204
C18:2 n-6	20.5 ± 2.6	20.4 ± 2.2	19.3 ± 2.9	0.436
C18:3 n-3	0.61 ± 0.09^a^	0.50 ± 0.11^b^	0.43 ± 0.10^b^	0.000
C20:3 n-6	0.87 ± 0.31	0.97 ± 0.33	0.95 ± 0.15	0.352
C20:4 n-6	8.52 ± 2.09	8.46 ± 1.74	7.10 ± 1.34	0.065
C22:6 n-3	0.92 ± 0.37	0.83 ± 0.34	0.67 ± 0.18	0.035

Data are means ± SD, *n* = 12–14 broilers/group. ^*^Only fatty acids > 0.5 g/100 g total fatty acids are shown

## Discussion

In the present study, the effect of replacement of soybean oil with HI larvae fat in broiler diets on the cecal microbiome and metabolic health was comprehensively investigated using omics technologies. Since no study existed in the literature reporting the lipid composition of HI fat in detail, with the exception of total lipid fatty acid composition, lipidomics was applied for the first time to fully characterize the HI fat. Our lipidomic analysis showed that the HI larvae fat used consisted almost completely (> 99%) of TG, whereas PG, PC, DG, PE and SM were only minor lipid classes. Amongst the TG species, saturated ones (34:0, 36:0, 38:0, 40:0, 42:0) made up approximately 60% of all TG species. TG species with one or two double bonds contributed to nearly 30% of all TG species, while those with three or more double bonds contributed less than 10% of all TG species. In line with the high proportion of saturated TG species and TG species with a low number of double bonds, additional fatty acid analysis of HI larvae fat total lipids revealed that C12:0, C16:0, C14:0 and C10:0 in decreasing order of their proportions made up nearly 80% of total fatty acids. Our observation that saturated TG species made up 60% of all TG species, while saturated fatty acids made up 80% of all fatty acids in HI fat total lipids, however, is not a discrepancy. This is likely attributed to the fact that saturated fatty acids are not exclusively found in TG species with zero double bonds, but are also present in TG species with 1 or more double bonds. Regardless of this, our data from lipidomics and fatty acid analysis clearly show that the HI fat used in the present study contained large amounts of saturated fatty acids, which largely explains the hard consistency of the HI fat at room temperature. Despite the marked difference in the fatty acid profile between soybean oil, the commonly used fat source in broiler diets, and HI larvae fat, partial or complete replacement of soybean oil with HI larvae fat had no effect on performance parameters (feed intake, body weight gain, feed:gain ratio) of the broilers over the whole 35 d-period. In line with the unaltered growth performance, ileal digestibility of ether extract determined during the finishing period did not differ among groups. This indicates that soybean oil replacement with HI larvae fat has neither beneficial nor adverse effects on fat digestibility, feed utilization and growth performance of broilers. However, our study revealed that feed intake and body weight gain, but not feed efficiency were increased by dietary inclusion of HI larvae fat during the starter period, whereas no effect was found during the grower and the finisher period. This suggests that feed acceptance was obviously improved in broilers during the early d of life by the use of HI larvae fat. The reason underlying this observation is unclear, but we exclude the possibility that the taste of the soybean oil used in the HI-0 diet was impaired due to fat deterioration during storage or diet preparation, because the same batch of soybean oil was also used for the diet of group HI-2.5, despite feed intake of this group was similar as in group HI-5.0. Unfortunately, comparable data from other studies are not available to explain our observation. While in two other studies dealing with the use of HI larvae fat as an alternative fat source in broiler diets performance data were reported either for the whole period [16] or only the finishing period [13], a further study did not provide any performance data [17]. However, the latter study is not suitable due to inadequate control of nutrient composition of the diets as evident from the marked difference of crude fat content between the starter diets (3.6% and 5.9% in the control diet and HI larva fat diet, respectively).

Despite studies demonstrating antimicrobial effects of MCFA in the intestine of broilers at dosages between 0.35% and 1.4% in the feed [22, 23], the present study, in which the dietary concentration of MCFA (sum of C10:0 and C12:0) was about 1.25% and 2.5% in groups HI-2.5 and HI-5.0, respectively, showed only a very little impact of dietary inclusion of HI larvae fat on the cecal microbiome. This was evident from the observation that cecal microbial diversity (α and β) did not differ between groups and taxonomic analysis revealed differences in the abundance of only four low-abundance bacterial taxa among groups; namely, the relative abundances of the phylum Actinobacteriota, the class Coriobacteriia, the order Coriobacteriales and the family Eggerthellaceae (all belonging to Actinobacteriota) were lower in group HI-5.0 compared to group HI-2.5, but the abundances of these bacterial taxa did not differ between groups HI-5.0 and HI-0 and between groups HI-2.5 and HI-0. No differences at all were found between groups in the abundances of bacterial families belonging to the two main phyla, Bacteroidota and Firmicutes, together accounting for approximately 99% of all bacteria in the three groups. Considering this and the rather small reduction in the abundances of the abovementioned bacterial taxa suggests that the impact of HI larvae fat on the cecal microbiota structure was very low. One important reason might be that the amount of MCFA reaching the cecum was not sufficient to exert pronounced antimicrobial effects. This assumption is supported by our observation that the ileal digestibility of crude fat was very high (approximately 90% in all groups) and the fact that TG-bound MCFA are hydrolysed and absorbed more rapidly than TG-bound long-chain fatty acids due to easier emulsification and less dependence on pancreatic lipase activity [27, 28]. In agreement with this, an increased ileal fat digestibility has been observed in a recent study with broilers by dietary substitution of soybean oil by HI fat [20]. Nevertheless, reports exist demonstrating that short-term administration of specific MCFA, such as caprylic acid (C8:0) and 1-monoglyceride of capric acid (C10:0), via the feed or the drinking water reduces cecal colonization of pathogenic bacteria, such as *Campylobacter jejuni*, after artificial infection [22, 23, 57]. However, contradictory results have been reported from another study, in which addition of either non-coated or coated specific MCFA (C6:0, C8:0, C10:0) to the feed at comparable concentrations as in the studies from Solis de los Santos et al. [22, 23] did not reduce cecal *Campylobacter* colonization in broilers [58]. The reason for these contradictory results is not clear, but differences between studies in the formulation of the MCFA in the feed may explain that the MCFA were less absorbed in the small intestine, thereby, reaching the ceca at higher concentrations in the one study than in the other. Thus, it is possible that the formulation of the diets containing HI larvae fat in the present study allowed an efficient absorption of MCFA in the small intestine, but a low passage of MCFA into the ceca of the broilers.

Owing to differences between bacterial families of different taxa with regard to the use of fermentation substrates and the metabolic pathways engaged in substrate utilization, a shift in the gut microbial community is typically accompanied by an altered profile of microbial fermentation products, such as SCFA, in the gut. Since dietary inclusion of HI larvae fat into the broiler diets did not cause a substantial shift in the bacterial community of the cecum, the unaltered concentrations of total and individual SCFA in the cecal digesta among the three groups are not surprising and are a further indication of the limited impact of HI larvae fat on the cecum microbiota of the broilers. In agreement with our results, a recent study demonstrated that complete replacement of 5% corn oil in the diet with HI larvae fat did not affect the concentrations of total and individual SCFA and viable counts of *Clostridium perfringens* in the ileum and cecum of broilers during a 30-d-feeding period [16]. In addition, feeding a diet containing about 1% MCFA as lauric acid had no influence on the DNA copy numbers of some beneficial bacteria (*Lactobacillus* and *Bifidobacterium* spp.), opportunistic pathogens (Enterobacteriaceae, *Escherichia coli*) and the pathogen *Campylobacter jejuni* in the jejunal digesta of broilers [59].

Consistent with our finding that the dietary fats including the HI larvae fat were readily absorbed in the small intestine, the present study revealed some effects of dietary inclusion of HI larvae fat on the hepatic and plasma lipidomes of the broilers. However, the effects of dietary inclusion of HI larvae fat were mainly related to the individual lipid species composition of the lipid classes, whereas the concentrations of the different lipid classes in liver and plasma were either not or only marginally (liver: PE, PG, LPC and LPE; plasma: CE and Cer) affected. In line with this, the sum of total lipids in liver and plasma were not influenced by dietary treatment. With regard to the individual lipid species composition of the different lipid classes, the most striking observation was that the proportions of lipid species with zero or few double bonds (e.g., in the liver: TG species with 0 and 1 double bonds, PC species with 1 and 3 double bonds, PE species with 2 and 3; in plasma: TG species with 0, 1 and 2 double bonds) were increased but those with four or more double bonds (e.g., in the liver: TG species with 5 and 6 double bonds, PC species with 4, 5 and 6 double bonds, PC species with 4, 6 and 8 double bonds; in plasma: TG species with 4, 5, 6, 7 and 8 double bonds) were decreased in group HI-5.0 compared to group HI-0. These alterations in the individual lipid species composition of lipid classes likely reflected the higher percentage of saturated fatty acids in the HI-5.0 broiler diets (59%–60%) than in the HI-2.5 (37%–38%) and the HI-0 (16%–17%) broiler diets, thereby, resulting in a higher intake of saturated fatty acids from HI larvae fat. Although most of the alterations in the plasma and liver lipidomes among the treatment groups were probably attributed to the marked difference in fatty acid composition between soybean oil and HI larvae fat, certain changes in the liver or plasma lipidomes might also reflect specific metabolic effects of the characteristic fatty acids, such as MCFA, contained in the HI larvae fat. For instance, the reason for the slight increase of CE by HI larvae fat might be based on the fact that MCFA, such as capric acid and lauric acid, and the long-chain saturated fatty acid myristic acid and palmitic acid exert hypercholesterolemic effects and increase blood levels of low-density lipoprotein (LDL) [60–62], in which CE makes up approximately 50%, at least in humans. Considering this, it appears possible that the raise in plasma CE levels in group HI-5.0 resulted from elevated LDL levels. The latter might be explained by an effect of HI larvae fat on different aspects of lipoprotein metabolism, such as the catabolism of very-LDL (VLDL) particles by lipoprotein lipase in extrahepatic tissues (white adipose tissue, skeletal muscle), hepatic clearance of LDL particles from plasma or the exchange of CE between lipoproteins which is governed by CE transfer protein.

In order to provide a deeper insight into the metabolic effects of HI larvae fat in broilers, differential transcriptome analysis of the liver was carried out. The finding that only 55 annotated genes out of more than 19,000 genes screened were found to be differentially expressed between group HI-5.0 and group HI-0 and the number of genes regulated greater twofold was very low, suggests that the impact of HI larvae fat inclusion on the hepatic transcriptome was modest. As expected, the impact of HI larvae fat inclusion on the hepatic transcriptome was less at the lower dietary inclusion level of HI larvae fat; i.e., only 25 annotated genes were identified as differentially expressed between groups HI-2.5 and HI-0, from which 7 genes were also differentially expressed between groups HI-5.0 and HI-0. Bioinformatic GSEA of these 55 differentially expressed hepatic genes revealed a particular involvement of the encoded proteins in biological processes dealing with sterol metabolism, such as cholesterol metabolic process, cholesterol biosynthetic process, sterol metabolic process, steroid biosynthetic process and lipid biosynthetic process. This observation was likely explained by the finding that several genes involved in sterol synthesis, such as *HMGCS1* (encoding 3-hydroxy-3-methylglutaryl-CoA synthase 1), *SCAP* (encoding SREBF chaperone), *AKR1D1* (encoding aldo–keto reductase family 1 member D1), *FDFT1* (encoding farnesyl-diphosphate farnesyltransferase 1) and *HSD17B7* (encoding hydroxysteroid 17-beta dehydrogenase 7), were amongst the genes differentially expressed between groups HI-5.0 or HI-2.5 and HI-0. Despite that the proteins encoded by *HMGCS1*, *FDFT1* and *SCAP* are involved in cholesterol synthesis [63], the unaltered levels of free cholesterol and CE in the liver between groups suggests that the regulation of these genes by HI larvae fat inclusion had no impact on hepatic cholesterol levels. Plausible reasons might be that the regulation of these genes occurred either only at the transcriptional level or at a too weak level to substantially alter the abundance of the encoded proteins. In any case, our findings from hepatic transcriptome analysis indicate that the effect of soybean oil replacement with HI larvae fat on intermediary metabolism of broilers is relatively weak.

## Conclusion

Comprehensive analysis of the effect of partial and complete replacement of soybean oil with HI larvae fat in broiler diets on growth performance, cecal microbiome, liver transcriptome and liver and plasma lipidomes revealed only very modest, but not any adverse effects of dietary HI larvae fat inclusion. Interestingly, growth performance of the broilers during the starter period was even improved by dietary inclusion of HI larvae fat. The findings of this study suggest that HI larvae fat can be used as an alternative fat source in broiler diets, which makes broiler production more sustainable through the exclusion of soybean oil and the utilization of HI larvae fat from regional production.

## Supplementary Information

**Additional file 1. Table S1** Characteristics of Gallus gallus gene-specific primers used for qPCR analysis. **Table S2** Concentrations of amino acids in the broiler diets. **Table S3** Operational taxonomic units (OTU) identified in cecum digesta of the broilers. **Table S4** List of differentially expressed transcripts in the liver of broilers between group HI-5.0 vs. HI-0. **Table S5** List of differentially expressed transcripts in the liver of broilers between group HI-2.5 vs. HI-0. **Table S6** qPCR validation of microarray data. **Table S7** Individual lipid species composition of triacylglycerols (TG), phosphatidylcholine (PC) and phosphatidylethanolamine (PE) in the liver of broilers fed diets with either 0% (HI-0), 2.5% (HI-2.5) or 5.0% (HI-5.0) Hermetia illucens (HI) larvae fat for 35 d. **Table S8** Individual lipid species composition of cholesteryl esters (CE), phosphatidylcholine (PC) and triacylglycerols (TG) in plasma of broilers fed diets with either 0% (HI-0), 2.5% (HI-2.5) or 5.0% (HI-5.0) Hermetia illucens (HI) larvae fat for 35 d.

## Data Availability

The datasets used and/or analysed during the current study are available from the corresponding author on reasonable request. The microarray data of this study have been deposited in MIAME compliant format in the NCBI´s Gene Expression Omnibus public repository.

## Abbreviations

AID: Apparent ileal digestibility
AKR1D1: Aldo–keto reductase family 1 member D1
CE: Cholesteryl esters
Cer: Ceramides
DG: Diglycerides
DM: Dry matter
FAME: Fatty acid methyl esters
FC: Free cholesterol
FDFT1: Farnesyl-diphosphate farnesyltransferase 1
GO: Gene ontology
GSEA: Gene set enrichment analysis
HI: *Hermetia illucens*
HMGCS1: 3-Hydroxy-3-methylglutaryl-CoA synthase 1
HSD17B7: Hydroxysteroid 17-beta dehydrogenase 7
LCFA: Long-chain fatty acids
LDL: Low-density lipoprotein
LPC: Lysophosphatidylcholines
LPE: Lysophosphatidylethanolamines
MCFA: Medium-chain fatty acids
NMDS: Non-metric multidimension distance scaling
OUT: Operational taxonomic units
PC: Phosphatidylcholines
PC O: Ether PC
PCA: Principal component analysis
PE: Phosphatidylethanolamines
PE O: Ether PE
PG: Phosphatidylglycerols
PI: Phosphatidylinositols
SCAP: SREBF chaperone
SCFA: Short-chain fatty acids
SFA: Saturated fatty acids
SM: Sphingomyelins
TG: Triglycerides
TM: Tenebrio molitor
VLDL: Very low-density lipoprotein
WPSA: World´s Poultry Science Association
