# Supplementary material for: Effect of replacement of soybean oil by Hermetia illucens fat on performance, digestibility, cecal microbiome, liver transcriptome and liver and plasma lipidomes of broilers
Source: J Anim Sci Biotechnol. 2023 Mar 1;14:20. doi: 10.1186/s40104-023-00831-6 (PMC9976384; doi:10.1186/s40104-023-00831-6)
Supplement: Supplementary file 1 — Additional file 1. Table S1 Characteristics of Gallus gallus gene-specific primers used for qPCR analysis. Table S2 Concentrations of amino acids in the broiler diets. Table S3 Operational taxonomic units (OTU) identified in cecum digesta of the broilers. Table S4 List of differentially expressed transcripts in the liver of broilers between group HI-5.0 vs. HI-0. Table S5 List of differentially expressed transcripts in the liver of broilers between group HI-2.5 vs. HI-0. Table S6 qPCR validation of microarray data. Table S7 Individual lipid species composition of triacylglycerols (TG), phosphatidylcholine (PC) and phosphatidylethanolamine (PE) in the liver of broilers fed diets with either 0% (HI-0), 2.5% (HI-2.5) or 5.0% (HI-5.0) Hermetia illucens (HI) larvae fat for 35 d. Table S8 Individual lipid species composition of cholesteryl esters (CE), phosphatidylcholine (PC) and triacylglycerols (TG) in plasma of broilers fed diets with either 0% (HI-0), 2.5% (HI-2.5) or 5.0% (HI-5.0) Hermetia illucens (HI) larvae fat for 35 d. [file 40104_2023_831_MOESM1_ESM.docx]

**Table S1** Characteristics of *Gallus gallus* gene-specific primers used for qPCR analysis

| **Gene symbol** | **Forward (5´ to 3´), Reverse (5´ to 3´)** | **PCR product size, bp** | **NCBI GenBank accession no.** |
| --- | --- | --- | --- |
| Reference genes |  |  |  |
| *ACTB* | ATGAAGCCCAGAGCAAAAGA, GGGGTGTTGAAGGTCTCAAA | 223 | NM_205518.1 |
| *GAPDH* | ACTGTCAAGGCTGAGAACGG, AGCTGAGGGAGCTGAGATGA | 204 | NM_204305.1 |
| *SDHA* | ATTCCCGTTTTGCCTACGGT**,** GGGAGTTTGCTCCAAGACGA | 172 | NM_001277398.1 |
| *YWHAZ* | TTCCAACTTCCGTCTGCCTC**,** AGCAGTCTTCCTCGCTTGAC | 328 | NM_001031343.1 |
| Target genes |  |  |  |
| *AKR1D1* | TTACTGTGGCAAGCTGTGGAA, GCTTGAAAGCCATTGGCAGC | 131 | NM_001277393 |
| *ANKRD22* | GGGATACTCTATTCGGAGCCAA, AAGGGGGTATCTCCACCGAA | 128 | NM_001277405 |
| *CA4* | CAGCCAAGCTGTGAAGACCC, TTTCAAAATGCAGCGGCGTC | 133 | XM_415893 |
| *DDO* | AGCACCCACTTTTGACTCGT, CTCTGCCAGGCCTCTTTGAA | 174 | XM_001234268 |
| *DPP4* | TGCTCACCGGCGAAAGTATC, CAAACCTCTGGTCAGGGGAC | 255 | NM_001031255 |
| *FANCL* | CAGTGGAGATGCGGTATGGC, GTGGAAGTCTCTACCCTGCG | 123 | NM_001033941 |
| *IL22RA2* | TGACGCCTATGAGCCCTACT, CCAGCACTCTGTGTTGCTCA | 284 | XM_001233761 |
| *HMGCS1* | CAGTTCTTGGGATGGACGCT, GCGGTCTAATGCACTGAGGT | 259 | NM_205411 |
| *MTHFS* | CTGCTGAGTCGCAAGGTGAT, ATAAGGTCAAGACCCCCGCC | 296 | NM_001277626 |
| *OGN* | CTGCAGCAACCTGGATGTAA, GGAGAATACTTTCTTTGTGACGTT | 158 | NM_204209 |
| *SCAP* | TATTTCACCCTTGTCCCGGC, GGCAACCGTTTGTTCAGGTC | 152 | XM_001231539 |
| *SH2D4A* | GCAAGGAGACCAGAGAGACAG, GTATTGCCTTCAGCGGGACT | 300 | XM_420452 |
| *SIK1* | GGCATTTGAGCGAAAGCGAA, CACCACTCCAAGGCTCCATA | 284 | NM_204682 |
| *SPATA4* | GGAAGAGAATCCACGACGGTT, GTTAATGCTGCAGCTCCGGT | 201 | NM_001031138 |
| *ULK1* | AAAGGCTCCTTTTCAGGCCA, CGCCAGGTGGGAAGTAGAAG | 289 | XM_415091 |
| *XDH* | CACGTTGCTTGTCCTGTTGC, GTTCCACATAGGCCCAGTTTTC | 234 | NM_205127 |

**Table S2** Concentrations of amino acids in the broiler diets

| **Amino acids, g/kg diet** | **Starter diets** | | | **Grower diets** | | | **Finisher diets** | | |
| --- | --- | --- | --- | --- | --- | --- | --- | --- | --- |
|  | **HI-0** | **HI-2.5** | **HI-5.0** | **HI-0** | **HI-2.5** | **HI-5.0** | **HI-0** | **HI-2.5** | **HI-5.0** |
| Alanine | 8.91 | 8.77 | 9.13 | 8.10 | 8.17 | 8.32 | 7.96 | 8.15 | 8.20 |
| Arginine | 14.29 | 14.34 | 13.45 | 12.97 | 13.28 | 13.49 | 12.10 | 12.23 | 12.32 |
| Asparagine | 18.85 | 18.40 | 18.76 | 16.36 | 16.72 | 16.90 | 16.10 | 16.28 | 16.40 |
| Cysteine | 4.02 | 3.90 | 4.04 | 3.58 | 3.68 | 3.73 | 3.54 | 3.49 | 3.93 |
| Glutamine | 37.80 | 37.61 | 38.17 | 35.42 | 35.75 | 36.01 | 35.54 | 35.88 | 36.11 |
| Glycine | 8.41 | 8.29 | 8.52 | 7.62 | 7.91 | 7.94 | 7.66 | 7.76 | 7.75 |
| Histidine | 4.91 | 4.85 | 4.92 | 4.39 | 4.67 | 4.63 | 4.44 | 4.46 | 4.58 |
| Isoleucine | 9.43 | 9.59 | 9.69 | 9.21 | 9.42 | 9.35 | 8.92 | 8.94 | 8.88 |
| Leucine | 15.86 | 15.83 | 16.18 | 14.79 | 14.92 | 15.05 | 14.49 | 14.74 | 14.89 |
| Lysine | 12.14 | 11.88 | 12.03 | 10.83 | 11.25 | 11.34 | 10.25 | 10.33 | 10.48 |
| Methionine | 5.98 | 6.07 | 5.92 | 5.56 | 5.57 | 5.50 | 5.14 | 5.17 | 5.18 |
| Phenylalanine | 9.86 | 10.06 | 10.00 | 9.03 | 9.25 | 9.35 | 8.99 | 9.10 | 9.19 |
| Proline | 12.86 | 12.78 | 13.17 | 11.24 | 11.62 | 11.54 | 11.75 | 11.54 | 11.31 |
| Serine | 10.55 | 10.26 | 10.26 | 9.31 | 9.49 | 9.64 | 9.30 | 9.25 | 9.47 |
| Threonine | 8.89 | 8.74 | 8.56 | 7.66 | 7.88 | 7.89 | 7.17 | 7.27 | 7.28 |
| Tryptophan | 2.53 | 2.54 | 2.48 | 2.21 | 2.37 | 2.32 | 2.16 | 1.97 | 2.22 |
| Tyrosine | 6.34 | 6.23 | 6.31 | 5.55 | 6.17 | 6.31 | 5.71 | 5.90 | 5.79 |
| Valine | 9.63 | 9.88 | 9.62 | 9.59 | 9.82 | 9.68 | 8.74 | 8.63 | 8.94 |

**Table S3** Operational taxonomic units (OTU) identified in cecum digesta of the broilers

|  | **Phylum** | **Class** | **Order** | **Family** | **Genus** |
| --- | --- | --- | --- | --- | --- |
| OTU2 | Firmicutes | Clostridia | Lachnospirales | Lachnospiraceae | *Ruminococcus* *torques* group |
| OTU5 | Firmicutes | Bacilli | Lactobacillales | Lactobacillaceae | *Lactobacillus* |
| OTU4 | Firmicutes | Bacilli | Lactobacillales | Lactobacillaceae | *Lactobacillus* |
| OTU15 | Firmicutes | Clostridia | Lachnospirales | Lachnospiraceae | |
| OTU12 | Firmicutes | Clostridia | Lachnospirales | Lachnospiraceae | *Blautia* |
| OTU14 | Firmicutes | Clostridia | Lachnospirales | Lachnospiraceae | |
| OTU8 | Firmicutes | Clostridia | Lachnospirales | Lachnospiraceae | |
| OTU6 | Firmicutes | Clostridia | Lachnospirales | Lachnospiraceae | |
| OTU7 | Firmicutes | Clostridia | Lachnospirales | Lachnospiraceae | |
| OTU9 | Firmicutes | Clostridia | Lachnospirales | Lachnospiraceae | *Eisenbergiella* |
| OTU3 | Firmicutes | Clostridia | Lachnospirales | Lachnospiraceae | |
| OTU11 | Firmicutes | Clostridia | Lachnospirales | Lachnospiraceae | *Ruminococcus* *torques* group |
| OTU13 | Firmicutes | Clostridia | Lachnospirales | Lachnospiraceae | |
| OTU27 | Firmicutes | Clostridia | Oscillospirales | Oscillospiraceae | *Oscillibacter* |
| OTU1 | Bacteroidota | Bacteroidia | Bacteroidales | Bacteroidaceae | *Bacteroides* |
| OTU18 | Firmicutes | Clostridia | Oscillospirales | Ruminococcaceae | |
| OTU22 | Firmicutes | Clostridia | Lachnospirales | Lachnospiraceae | |
| OTU10 | Bacteroidota | Bacteroidia | Bacteroidales | Bacteroidaceae | *Bacteroides* |
| OTU17 | Firmicutes | Clostridia | Lachnospirales | Lachnospiraceae | *Anaerostipes* |
| OTU19 | Firmicutes | Clostridia | Lachnospirales | Lachnospiraceae | *Sellimonas* |
| OTU23 | Firmicutes | Clostridia | Oscillospirales | Eubacterium coprostanoligenes group | |
| OTU16 | Firmicutes | Clostridia | Oscillospirales | Oscillospiraceae | *Colidextribacter* |
| OTU21 | Firmicutes | Clostridia | Lachnospirales | Lachnospiraceae | |
| OTU20 | Firmicutes | Clostridia | Lachnospirales | Lachnospiraceae | |
| OTU26 | Firmicutes | Clostridia | Lachnospirales | Lachnospiraceae | *Eisenbergiella* |
| OTU24 | Firmicutes | Clostridia | Oscillospirales | Butyricicoccaceae | *Butyricicoccus* |
| OTU25 | Firmicutes | Clostridia | Monoglobales | Monoglobaceae | *Monoglobus* |
| OTU44 | Firmicutes | Bacilli | Erysipelotrichales | Erysipelotrichaceae | *Turicibacter* |
| OTU31 | Firmicutes | Clostridia | Peptostreptococcales-Tissierellales | Peptostreptococcaceae | |
| OTU43 | Firmicutes | Bacilli | Erysipelotrichales | Erysipelotrichaceae | |
| OTU33 | Firmicutes | Bacilli | Erysipelotrichales | Erysipelatoclostridiaceae | *Erysipelatoclostridium* |
| OTU47 | Firmicutes | Clostridia | Lachnospirales | Lachnospiraceae | |
| OTU102 | Firmicutes | Clostridia | Peptostreptococcales-Tissierellales | Peptostreptococcaceae | *Romboutsia* |
| OTU30 | Proteobacteria | Gammaproteobacteria | Enterobacterales | Enterobacteriaceae | *Escherichia-Shigella* |
| OTU53 | Firmicutes | Clostridia | Lachnospirales | Lachnospiraceae | |
| OTU29 | Firmicutes | Clostridia | Lachnospirales | Lachnospiraceae | |
| OTU35 | Firmicutes | Clostridia | Lachnospirales | Lachnospiraceae | *Anaerostipes* |
| OTU34 | Firmicutes | Clostridia | Clostridia UCG-014 | |  |
| OTU28 | Firmicutes | Clostridia | Lachnospirales | Lachnospiraceae | |
| OTU36 | Firmicutes | Clostridia | Lachnospirales | Lachnospiraceae | |
| OTU49 | Firmicutes | Clostridia | Oscillospirales | Oscillospiraceae | *Colidextribacter* |
| OTU37 | Firmicutes | Clostridia | Oscillospirales | Butyricicoccaceae | *Butyricicoccus* |
| OTU105 | Firmicutes | Clostridia | Lachnospirales | Lachnospiraceae | |
| OTU46 | Firmicutes | Clostridia | Lachnospirales | Lachnospiraceae | |
| OTU61 | Firmicutes | Bacilli | Erysipelotrichales | Erysipelatoclostridiaceae | *Erysipelatoclostridium* |
| OTU32 | Firmicutes | Clostridia | Lachnospirales | Lachnospiraceae | *Eubacterium hallii group* |
| OTU50 | Firmicutes | Clostridia | Lachnospirales | Lachnospiraceae | GCA-900066575 |
| OTU73 | Firmicutes | Bacilli | Erysipelotrichales | Erysipelotrichaceae | |
| OTU59 | Firmicutes | Clostridia | Oscillospirales | Ruminococcaceae | |
| OTU39 | Firmicutes | Clostridia | Oscillospirales | Ruminococcaceae | *Incertae Sedis* |
| OTU54 | Firmicutes | Clostridia | Lachnospirales | Lachnospiraceae | *Blautia* |
| OTU52 | Firmicutes | Clostridia | Oscillospirales | Oscillospiraceae | |
| OTU40 | Firmicutes | Clostridia | Lachnospirales | Lachnospiraceae | *Ruminococcus* *torques* group |
| OTU42 | Firmicutes | Clostridia | Lachnospirales | Lachnospiraceae | *Eubacterium hallii* group |
| OTU60 | Firmicutes | Clostridia | Lachnospirales | Lachnospiraceae | *Blautia* |
| OTU38 | Firmicutes | Clostridia | Oscillospirales | Ruminococcaceae | *Incertae Sedis* |
| OTU48 | Firmicutes | Clostridia | Lachnospirales | Lachnospiraceae | *Eubacterium hallii* group |
| OTU70 | Firmicutes | Clostridia | Lachnospirales | Lachnospiraceae | |
| OTU45 | Firmicutes | Clostridia | Lachnospirales | Lachnospiraceae | *Eubacterium hallii* group |
| OTU57 | Firmicutes | Clostridia | Oscillospirales | Oscillospiraceae | |
| OTU66 | Firmicutes | Bacilli | Erysipelotrichales | Erysipelotrichaceae | *Clostridium innocuum* group |
| OTU41 | Firmicutes | Clostridia | Oscillospirales | Ruminococcaceae | *Incertae Sedis* |
| OTU72 | Actinobacteriota | Coriobacteriia | Coriobacteriales | Eggerthellaceae |  |
| OTU65 | Firmicutes | Clostridia | Lachnospirales | Lachnospiraceae | *Anaerostipes* |
| OTU77 | Firmicutes | Clostridia | Lachnospirales | Lachnospiraceae | *Lachnoclostridium* |
| OTU58 | Firmicutes | Clostridia | Oscillospirales | Ruminococcaceae | *Incertae Sedis* |
| OTU69 | Firmicutes | Clostridia | Oscillospirales | Ruminococcaceae | |
| OTU67 | Firmicutes | Clostridia | Lachnospirales | Lachnospiraceae | |
| OTU82 | Firmicutes | Clostridia | Oscillospirales | Butyricicoccaceae | UCG-009 |
| OTU55 | Firmicutes | Clostridia | Lachnospirales | Lachnospiraceae | |
| OTU62 | Firmicutes | Clostridia | Oscillospirales | Ruminococcaceae | |
| OTU63 | Firmicutes | Clostridia | Lachnospirales | Lachnospiraceae | |
| OTU64 | Firmicutes | Clostridia | Lachnospirales | Lachnospiraceae | |
| OTU81 | Firmicutes | Clostridia | Lachnospirales | Lachnospiraceae | |
| OTU68 | Firmicutes | Clostridia | Oscillospirales | Ruminococcaceae | |
| OTU80 | Firmicutes | Clostridia | Lachnospirales | Lachnospiraceae | |
| OTU74 | Firmicutes | Clostridia | Oscillospirales | Ruminococcaceae | *Anaerotruncus* |
| OTU93 | Firmicutes | Clostridia | Oscillospirales | Oscillospiraceae | *Flavonifractor* |
| OTU87 | Firmicutes | Clostridia | Oscillospirales | Ruminococcaceae | |
| OTU75 | Firmicutes | Clostridia | Oscillospirales | Ruminococcaceae | DTU089 |
| OTU97 | Firmicutes | Clostridia | Lachnospirales | Lachnospiraceae | *Ruminococcus torques* group |
| OTU86 | Firmicutes | Clostridia | Oscillospirales | Oscillospiraceae | *Flavonifractor* |
| OTU78 | Firmicutes | Clostridia | Peptostreptococcales-Tissierellales | Peptostreptococcaceae | *Clostridioides* |
| OTU134 | Firmicutes | Clostridia | Oscillospirales | Oscillospiraceae | *Oscillibacter* |
| OTU56 | Firmicutes | Clostridia | Lachnospirales | Lachnospiraceae | |
| OTU96 | Firmicutes | Bacilli | Erysipelotrichales | Erysipelatoclostridiaceae | *Erysipelatoclostridium* |
| OTU51 | Firmicutes | Clostridia | Lachnospirales | Lachnospiraceae | *Ruminococcus gnavus* group |
| OTU71 | Firmicutes | Clostridia | Oscillospirales | Ruminococcaceae | *Incertae Sedis* |
| OTU89 | Firmicutes | Clostridia | Lachnospirales | Lachnospiraceae | *Shuttleworthia* |
| OTU131 | Firmicutes | Clostridia | Clostridiales | Clostridiaceae | *Clostridium* sensu stricto 1 |

**Table S4** List of differentially expressed transcripts in the liver of broilers between group HI-5.0 vs. HI-0

| **Gene Symbol** | **HI-5.0 vs. HI-0** | | |
| --- | --- | --- | --- |
|  | **Sig log ratio** | **Fold change** | ***P*-value** |
| *BDKRB1* | 1.36 | 2.57 | 0.0222 |
| *XDH* | 1.24 | 2.36 | 0.0013 |
| *IGJ* | 1.07 | 2.10 | 0.0142 |
| *DPP4* | 0.92 | 1.90 | 0.0035 |
| *ENDOUL* | 0.87 | 1.83 | 0.0030 |
| *UPP2* | 0.86 | 1.82 | 0.0321 |
| *EMB* | 0.86 | 1.81 | 0.0008 |
| *BASP1* | 0.81 | 1.76 | 0.0266 |
| *SIK1* | 0.78 | 1.71 | 0.0347 |
| *B3GALT2* | 0.77 | 1.71 | 0.0095 |
| *MMP1* | 0.76 | 1.70 | 0.0438 |
| *SH2D4A* | 0.74 | 1.67 | 0.0232 |
| *OGN* | 0.73 | 1.66 | 0.0010 |
| *PAPLN* | 0.73 | 1.66 | 0.0235 |
| *HMGCS1* | 0.70 | 1.63 | 0.0272 |
| *C5H11ORF96* | 0.70 | 1.62 | 0.0349 |
| *C7* | 0.67 | 1.60 | 0.0007 |
| *AKR1D1* | 0.67 | 1.59 | 0.0473 |
| *F13A1* | 0.66 | 1.58 | 0.0230 |
| *GAR1* | 0.66 | 1.58 | 0.0199 |
| *CDH11* | 0.65 | 1.57 | 0.0090 |
| *MYLK* | 0.64 | 1.55 | 0.0312 |
| *FAM150B* | 0.63 | 1.55 | 0.0253 |
| *LOC770434* | 0.63 | 1.55 | 0.0412 |
| *GUCY1A2* | 0.62 | 1.54 | 0.0451 |
| *EMCN* | 0.61 | 1.53 | 0.0188 |
| *KCNK5* | 0.61 | 1.53 | 0.0043 |
| *PIKFYVE* | 0.60 | 1.52 | 0.0406 |
| *F2RL2* | 0.60 | 1.52 | 0.0118 |
| *LOC427201* | 0.60 | 1.51 | 0.0040 |
| *ZBTB16* | 0.59 | 1.51 | 0.0171 |
| *KLF10* | -0.60 | -1.52 | 0.0194 |
| *MTHFS* | -0.61 | -1.53 | 0.0035 |
| *WDR66* | -0.62 | -1.54 | 0.0056 |
| *RIPK3* | -0.62 | -1.54 | 0.0229 |
| *HSD17B7* | -0.62 | -1.54 | 0.0484 |
| *XKR9* | -0.63 | -1.55 | 0.0051 |
| *PISD* | -0.64 | -1.56 | 0.0493 |
| *DNAJC6* | -0.65 | -1.56 | 0.0136 |
| *SLC4A10* | -0.65 | -1.57 | 0.0188 |
| *SLC25A4* | -0.67 | -1.59 | 0.0240 |
| *OSGIN1* | -0.77 | -1.70 | 0.0166 |
| *SLC19A2* | -0.77 | -1.71 | 0.0106 |
| *SLC22A16* | -0.81 | -1.75 | 0.0342 |
| *FANCL* | -0.82 | -1.76 | 0.0218 |
| *ULK1* | -0.85 | -1.81 | 0.0358 |
| *CA4* | -0.86 | -1.82 | 0.0446 |
| *SPATA4* | -0.88 | -1.84 | 0.0256 |
| *SCAP* | -0.91 | -1.88 | 0.0175 |
| *DDO* | -0.92 | -1.89 | 0.0119 |
| *SLC6A13* | -1.08 | -2.12 | 0.0066 |
| *ATP2B2* | -1.11 | -2.15 | 0.0004 |
| *LOC101749538* | -1.35 | -2.55 | 0.0396 |
| *ANKRD22* | -1.91 | -3.76 | 0.0183 |
| *IL22RA2* | -2.35 | -5.09 | 0.0014 |

**Table S5** List of differentially expressed transcripts in the liver of broilers between group HI-2.5 vs. HI-0

| **Gene symbol** | **HI-2.5 vs. HI-0** | | |
| --- | --- | --- | --- |
|  | **Sig log ratio** | **Fold change** | ***P*-value** |
| *NSMF* | 1.19 | 2.29 | 0.0057 |
| *CHIA* | 1.11 | 2.16 | 0.0397 |
| *BDKRB1* | 1.03 | 2.04 | 0.0006 |
| *ENPEP* | 0.85 | 1.80 | 0.0294 |
| *PFKFB3* | 0.85 | 1.80 | 0.0357 |
| *MIR1790* | 0.78 | 1.72 | 0.0487 |
| *SIK1* | 0.78 | 1.71 | 0.0278 |
| *DPP4* | 0.77 | 1.71 | 0.0226 |
| *LOC395159* | 0.76 | 1.69 | 0.0484 |
| *CP* | 0.73 | 1.66 | 0.0323 |
| *DKK3* | 0.69 | 1.61 | 0.0194 |
| *AKR1D1* | 0.66 | 1.59 | 0.0215 |
| *CDC42EP3* | 0.65 | 1.57 | 0.0007 |
| *FABP5* | 0.65 | 1.57 | 0.0368 |
| *IQSEC3* | 0.65 | 1.57 | 0.0137 |
| *TLR1LA* | 0.64 | 1.56 | 0.0468 |
| *PLACL2* | 0.62 | 1.54 | 0.0493 |
| *MIR1587* | 0.61 | 1.53 | 0.0188 |
| *DCDC2* | 0.59 | 1.51 | 0.0160 |
| *PROM1* | -0.59 | -1.50 | 0.0127 |
| *DDO* | -0.59 | -1.51 | 0.0465 |
| *MIR17* | -0.59 | -1.51 | 0.0282 |
| *INDOL1* | -0.60 | -1.52 | 0.0023 |
| *ATP2B2* | -0.68 | -1.60 | 0.0085 |
| *GUCY2C* | -0.77 | -1.70 | 0.0236 |

**Table S6** qPCR validation of microarray data

| **Gene symbol** | **FC** | | ***P*-value** | |
| --- | --- | --- | --- | --- |
|  | **Microarray** | **qPCR** | **Microarray** | **qPCR** |
| *AKR1D1* | 1.66 | 1.58 | 0.039 | 0.067 |
| *ANKRD22* | -1.99 | -2.23 | 0.018 | 0.059 |
| *CA4* | -1.92 | -2.15 | 0.022 | 0.034 |
| *DDO* | -1.6 | -2.63 | 0.007 | 0.003 |
| *DPP4* | 1.97 | 1.32 | 0.005 | 0.255 |
| *FANCL* | -2.05 | -1.50 | 0.023 | 0.169 |
| *IL22RA2* | -5.37 | -5.27 | 0.001 | 0.009 |
| *HMGCS1* | 1.85 | 3.36 | 0.011 | 0.003 |
| *MTHFS* | -1.54 | -1.35 | 0.002 | 0.054 |
| *OGN* | 1.61 | 1.30 | 0.007 | 0.066 |
| *SCAP* | -1.88 | -2.06 | 0.004 | 0.016 |
| *SH2D4A* | 1.53 | 1.74 | 0.013 | 0.021 |
| *SIK1* | 1.89 | 1.37 | 0.022 | 0.161 |
| *SPATA4* | -2.03 | -1.71 | 0.010 | 0.199 |
| *ULK1* | -1.97 | -2.16 | 0.027 | 0.013 |
| *XDH* | 2.60 | 2.52 | 0.001 | 0.013 |

**Table S7** Individual lipid species composition of triacylglycerols (TG), phosphatidylcholine (PC) and phosphatidylethanolamine (PE) in the liver of broilers fed diets with either 0% (HI-0), 2.5% (HI-2.5) or 5.0% (HI-5.0) *Hermetia illucens* (HI) larvae fat for 35 d

| **Fatty acid, % of total species** | **HI-0** |  | **HI-2.5** |  | **HI-5.0** |  | ***P*-value** |
| --- | --- | --- | --- | --- | --- | --- | --- |
| TG species |  | | | | | |  |
| 40:0 | 0.005 ± 0.010 | ^c^ | 0.065 ± 0.037 | ^b^ | 0.123 ± 0.087 | ^a^ | <0.001 |
| 40:1 | 0.003 ± 0.003 | ^c^ | 0.013 ± 0.007 | ^b^ | 0.044 ± 0.020 | ^a^ | <0.001 |
| 42:0 | 0.001 ± 0.003 | ^c^ | 0.069 ± 0.027 | ^b^ | 0.188 ± 0.113 | ^a^ | <0.001 |
| 42:1 | 0.008 ± 0.019 | ^b^ | 0.030 ± 0.052 | ^b^ | 0.088 ± 0.060 | ^a^ | <0.001 |
| 42:2 | 0.004 ± 0.005 | ^b^ | 0.123 ± 0.079 | ^a^ | 0.138 ± 0.107 | ^a^ | <0.001 |
| 44:0 | 0.010 ± 0.007 | ^c^ | 0.230 ± 0.081 | ^b^ | 0.523 ± 0.233 | ^a^ | <0.001 |
| 44:1 | 0.019 ± 0.011 | ^c^ | 0.198 ± 0.051 | ^b^ | 0.552 ± 0.222 | ^a^ | <0.001 |
| 44:2 | 0.012 ± 0.006 | ^c^ | 0.130 ± 0.040 | ^b^ | 0.239 ± 0.112 | ^a^ | <0.001 |
| 44:3 | 0.003 ± 0.002 | ^c^ | 0.025 ± 0.009 | ^b^ | 0.045 ± 0.020 | ^a^ | <0.001 |
| 46:0 | 0.141 ± 0.057 | ^c^ | 0.594 ± 0.224 | ^b^ | 1.010 ± 0.440 | ^a^ | <0.001 |
| 46:1 | 0.157 ± 0.048 | ^c^ | 1.010 ± 0.250 | ^b^ | 2.160 ± 0.630 | ^a^ | <0.001 |
| 46:2 | 0.072 ± 0.021 | ^b^ | 0.686 ± 0.188 | ^a^ | 1.170 ± 0.350 | ^a^ | <0.001 |
| 46:3 | 0.006 ± 0.006 | ^c^ | 0.137 ± 0.039 | ^b^ | 0.239 ± 0.085 | ^a^ | <0.001 |
| 47:1 | 0.001 ± 0.002 | ^c^ | 0.048 ± 0.0159 | ^b^ | 0.103 ± 0.032 | ^a^ | <0.001 |
| 48:0 | 0.894 ± 0.372 | ^b^ | 1.340 ± 0.450 | ^a^ | 1.700 ± 0.540 | ^a^ | 0.001 |
| 48:1 | 1.420 ± 0.520 | ^c^ | 3.230 ± 0.860 | ^b^ | 4.830 ± 1.460 | ^a^ | <0.001 |
| 48:2 | 0.845 ± 0.257 | ^c^ | 2.550 ± 0.570 | ^b^ | 3.530 ± 0.910 | ^a^ | <0.001 |
| 48:3 | 0.207 ± 0.040 | ^b^ | 0.900 ± 0.204 | ^a^ | 1.110 ± 0.280 | ^a^ | <0.001 |
| 49:0 | 0.012 ± 0.009 | ^b^ | 0.034 ± 0.019 | ^a^ | 0.048 ± 0.017 | ^a^ | <0.001 |
| 49:1 | 0.045 ± 0.016 | ^c^ | 0.106 ± 0.025 | ^b^ | 0.166 ± 0.028 | ^a^ | <0.001 |
| 49:2 | 0.038 ± 0.016 | ^c^ | 0.101 ± 0.027 | ^b^ | 0.142 ± 0.024 | ^a^ | <0.001 |
| 49:3 | 0.010 ± 0.007 | ^c^ | 0.046 ± 0.012 | ^b^ | 0.059 ± 0.012 | ^a^ | <0.001 |
| 50:0 | 1.490 ± 0.590 |  | 1.690 ± 0.600 |  | 1.660 ± 0.600 |  | 0.676 |
| 50:1 | 7.730 ± 2.660 |  | 8.280 ± 2.110 |  | 9.470 ± 2.140 |  | 0.179 |
| 50:2 | 6.250 ± 1.670 | ^b^ | 7.330 ± 1.600 | ^ab^ | 8.590 ± 0.890 | ^a^ | 0.001 |
| 50:3 | 1.990 ± 0.304 | ^b^ | 2.650 ± 0.420 | ^a^ | 3.160 ± 0.730 | ^a^ | <0.001 |
| 50:4 | 0.390 ± 0.102 | ^b^ | 0.594 ± 0.120 | ^a^ | 0.718 ± 0.340 | ^a^ | <0.001 |
| 50:5 | 0.084 ± 0.049 | ^b^ | 0.143 ± 0.050 | ^a^ | 0.182 ± 0.129 | ^ab^ | 0.008 |
| 50:6 | 0.007 ± 0.012 | ^b^ | 0.028 ± 0.017 | ^a^ | 0.049 ± 0.050 | ^a^ | <0.001 |
| 51:1 | 0.126 ± 0.024 | ^b^ | 0.172 ± 0.033 | ^b^ | 0.194 ± 0.028 | ^a^ | <0.001 |
| 51:2 | 0.175 ± 0.016 | ^b^ | 0.229 ± 0.03 | ^b^ | 0.248 ± 0.023 | ^a^ | <0.001 |
| 51:3 | 0.104 ± 0.016 | ^b^ | 0.132 ± 0.024 | ^a^ | 0.131 ± 0.032 | ^a^ | 0.014 |
| 52:1 | 8.580 ± 2.820 |  | 7.780 ± 1.700 |  | 6.760 ± 2.180 |  | 0.154 |
| 52:2 | 22.6 ± 4.70 | ^a^ | 20.4 ± 3.5 | ^ab^ | 18.6 ± 3.0 | ^b^ | 0.042 |
| 52:3 | 15.6 ± 1.6 | ^a^ | 13.7 ± 1.7 | ^b^ | 11.3 ± 2.5 | ^c^ | <0.001 |
| 52:4 | 4.390 ± 1.270 | ^a^ | 3.550 ± 0.830 | ^ab^ | 2.950 ± 1.510 | ^b^ | 0.021 |
| 52:5 | 0.953 ± 0.459 |  | 0.758 ± 0.256 |  | 0.766 ± 0.614 |  | 0.494 |
| 52:6 | 0.179 ± 0.126 |  | 0.147 ± 0.058 |  | 0.188 ± 0.186 |  | 0.920 |
| 53:1 | 0.075 ± 0.028 |  | 0.091 ± 0.032 |  | 0.089 ± 0.019 |  | 0.154 |
| 53:2 | 0.202 ± 0.026 | ^b^ | 0.234 ± 0.027 | ^a^ | 0.222 ± 0.025 | ^ab^ | 0.012 |
| 53:3 | 0.180 ± 0.058 |  | 0.184 ± 0.047 |  | 0.153 ± 0.036 |  | 0.235 |
| 53:4 | 0.164 ± 0.036 | ^a^ | 0.162 ± 0.040 | ^a^ | 0.127 ± 0.022 | ^b^ | 0.002 |
| 54:2 | 3.490 ± 0.650 |  | 3.260 ± 0.740 |  | 2.870 ± 0.620 |  | 0.094 |
| 54:3 | 5.890 ± 1.430 | ^a^ | 5.080 ± 1.290 | ^ab^ | 4.520 ± 1.190 | ^b^ | 0.047 |
| 54:4 | 4.710 ± 1.880 | ^a^ | 3.670 ± 1.220 | ^ab^ | 3.040 ± 1.400 | ^b^ | 0.033 |
| 54:5 | 3.190 ± 1.800 | ^a^ | 2.270 ± 1.000 | ^a^ | 1.640 ± 1.010 | ^b^ | 0.002 |
| 54:6 | 1.640 ± 1.130 | ^a^ | 1.140 ± 0.630 | ^ab^ | 0.761 ± 0.564 | ^b^ | 0.003 |
| 54:7 | 0.408 ± 0.333 |  | 0.238 ± 0.156 |  | 0.193 ± 0.195 |  | 0.081 |
| 55:2 | 0.043 ± 0.016 |  | 0.048 ± 0.016 |  | 0.049 ± 0.012 |  | 0.419 |
| 55:3 | 0.059 ± 0.018 |  | 0.066 ± 0.023 |  | 0.063 ± 0.016 |  | 0.584 |
| 55:4 | 0.035 ± 0.015 |  | 0.0394 ± 0.015 |  | 0.034 ± 0.016 |  | 0.590 |
| 56:1 | 0.081 ± 0.024 |  | 0.090 ± 0.035 |  | 0.084 ± 0.011 |  | 0.953 |
| 56:2 | 0.218 ± 0.035 |  | 0.225 ± 0.072 |  | 0.202 ± 0.030 |  | 0.470 |
| 56:3 | 0.337 ± 0.092 | ^a^ | 0.328 ± 0.099 |  | 0.287 ± 0.061 |  | 0.049 |
| 56:4 | 0.542 ± 0.248 |  | 0.509 ± 0.226 |  | 0.348 ± 0.112 |  | 0.070 |
| 56:5 | 1.090 ± 0.760 |  | 0.914 ± 0.587 |  | 0.592 ± 0.289 |  | 0.134 |
| 56:6 | 1.300 ± 1.180 |  | 0.941 ± 0.752 |  | 0.647 ± 0.398 |  | 0.219 |
| 56:7 | 0.689 ± 0.692 |  | 0.457 ± 0.420 |  | 0.348 ± 0.289 |  | 0.301 |
| 56:8 | 0.318 ± 0.335 |  | 0.194 ± 0.177 |  | 0.140 ± 0.128 |  | 0.198 |
| 58:2 | 0.035 ± 0.014 |  | 0.041 ± 0.023 |  | 0.040 ± 0.011 |  | 0.900 |
| 58:3 | 0.043 ± 0.019 |  | 0.038 ± 0.019 |  | 0.032 ± 0.007 |  | 0.139 |
| 58:5 | 0.125 ± 0.084 |  | 0.110 ± 0.047 |  | 0.074 ± 0.027 |  | 0.089 |
| 58:6 | 0.178 ± 0.156 |  | 0.150 ± 0.106 |  | 0.102 ± 0.056 |  | 0.336 |
| 58:7 | 0.190 ± 0.188 |  | 0.145 ± 0.128 |  | 0.106 ± 0.078 |  | 0.318 |
| 58:8 | 0.178 ± 0.194 |  | 0.126 ± 0.132 |  | 0.087 ± 0.077 |  | 0.280 |
|  |  |  |  |  |  |  |  |
| Sum C40 | 0.01 ± 0.01 | ^c^ | 0.08 ± 0.04 | ^b^ | 0.17 ± 0.11 | ^a^ | <0.001 |
| Sum C42 | 0.01 ± 0.02 | ^c^ | 0.22 ± 0.13 | ^b^ | 0.41 ± 0.26 | ^a^ | <0.001 |
| Sum C44 | 0.04 ± 0.02 | ^c^ | 0.58 ± 0.16 | ^b^ | 1.36 ± 0.58 | ^a^ | <0.001 |
| Sum C46 | 0.38 ± 0.11 | ^b^ | 2.43 ± 0.61 | ^a^ | 4.58 ± 1.37 | ^a^ | <0.001 |
| Sum C47 | 0.01 ± 0.01 | ^c^ | 0.05 ± 0.02 | ^b^ | 0.10 ± 0.03 | ^a^ | <0.001 |
| Sum C48 | 3.36 ± 1.12 | ^c^ | 8.02 ± 1.81 | ^b^ | 11.2 ± 2.9 | ^a^ | <0.001 |
| Sum C49 | 0.10 ± 0.04 | ^c^ | 0.29 ± 0.08 | ^b^ | 0.41 ± 0.06 | ^a^ | <0.001 |
| Sum C50 | 17.9 ± 5.0 | ^b^ | 20.7 ± 4.0 | ^ab^ | 23.8 ± 2.5 | ^a^ | 0.004 |
| Sum C51 | 0.41 ± 0.04 | ^b^ | 0.53 ± 0.07 | ^a^ | 0.57 ± 0.05 | ^a^ | <0.001 |
| Sum C52 | 52.3 ± 4.5 | ^a^ | 46.3 ± 3.3 | ^b^ | 40.5 ± 3.0 | ^c^ | <0.001 |
| Sum C53 | 0.62 ± 0.09 |  | 0.67 ± 0.11 |  | 0.59 ± 0.07 |  | 0.104 |
| Sum C54 | 19.3 ± 6.3 | ^a^ | 15.7 ± 4.4 | ^ab^ | 13.0 ± 4.1 | ^b^ | 0.034 |
| Sum C55 | 0.14 ± 0.03 |  | 0.15 ± 0.03 |  | 0.15 ± 0.03 |  | 0.409 |
| Sum C56 | 4.57 ± 3.24 | ^a^ | 3.66 ± 2.32 | ^ab^ | 2.65 ± 1.24 | ^b^ | 0.019 |
| Sum C58 | 0.75 ± 0.62 |  | 0.61 ± 0.45 |  | 0.44 ± 0.24 |  | 0.290 |
|  |  |  |  |  |  |  |  |
| PC species |  |  |  |  |  |  |  |
| 32:0 | 2.34 ± 0.36 |  | 2.70 ± 0.45 |  | 2.53 ± 0.29 |  | 0.073 |
| 32:1 | 0.60 ± 0.19 | ^b^ | 0.96 ± 0.24 | ^b^ | 1.58 ± 0.47 | ^a^ | <0.001 |
| 34:1 | 10.2 ± 2.0 | ^b^ | 11.5 ± 2.0 | ^ab^ | 13.1 ± 2.0 | ^a^ | 0.003 |
| 34:2 | 17.9 ± 1.3 |  | 19.0 ± 1.7 |  | 18.4 ± 1.1 |  | 0.194 |
| 34:3 | 1.13 ± 0.24 |  | 1.12 ± 0.15 |  | 1.35 ± 0.28 |  | 0.478 |
| 34:4 | 0.10 ± 0.03 | ^c^ | 0.15 ± 0.02 | ^b^ | 0.21 ± 0.04 | ^a^ | <0.001 |
| 36:1 | 4.00 ± 0.71 | ^b^ | 4.17 ± 0.88 | ^b^ | 5.69 ± 0.96 | ^a^ | <0.001 |
| 36:2 | 27.3 ± 3.0 |  | 26.8 ± 1.9 |  | 27.0 ± 1.8 |  | 0.855 |
| 36:3 | 3.99 ± 0.61 | ^b^ | 4.14 ± 0.62 | ^b^ | 4.88 ± 0.38 | ^a^ | <0.001 |
| 36:4 | 8.34 ± 1.47 | ^a^ | 7.86 ± 0.94 | ^a^ | 6.66 ± 1.03 | ^b^ | 0.003 |
| 36:5 | 0.80 ± 0.19 |  | 0.70 ± 0.12 |  | 0.70 ± 0.14 |  | 0.182 |
| 38:2 | 0.43 ± 0.08 |  | 0.43 ± 0.07 |  | 0.50 ± 0.10 |  | 0.099 |
| 38:3 | 2.27 ± 0.38 |  | 2.30 ± 0.38 |  | 2.59 ± 0.32 |  | 0.063 |
| 38:4 | 11.9 ± 3.0 | ^a^ | 10.6 ± 2.2 | ^ab^ | 8.41 ± 2.05 | ^b^ | 0.003 |
| 38:5 | 3.19 ± 0.73 | ^a^ | 2.84 ± 0.47 | ^ab^ | 2.57 ± 0.48 | ^b^ | 0.034 |
| 38:6 | 2.62 ± 0.60 | ^a^ | 2.30 ± 0.51 | ^b^ | 1.71 ± 0.35 | ^b^ | <0.001 |
| 40:4 | 0.54 ± 0.08 |  | 0.49 ± 0.06 |  | 0.44 ± 0.06 |  | 0.054 |
| 40:5 | 1.01 ± 0.22 | ^a^ | 0.88 ± 0.12 | ^ab^ | 0.78 ± 0.15 | ^b^ | 0.008 |
| 40:6 | 1.16 ± 0.26 | ^a^ | 1.01 ± 0.24 | ^ab^ | 0.80 ± 0.18 | ^b^ | 0.001 |
| 40:7 | 0.16 ± 0.03 |  | 0.13 ± 0.03 |  | 0.14 ± 0.04 |  | 0.098 |
|  |  |  |  |  |  |  |  |
| Sum C32 | 2.97 ± 0.50 | ^b^ | 3.61 ± 0.59 | ^b^ | 4.11 ± 0.66 | ^a^ | <0.001 |
| Sum C34 | 29.3 ± 2.6 | ^a^ | 31.7 ± 2.3 | ^b^ | 33.1 ± 2.3 | ^a^ | 0.002 |
| Sum C36 | 44.4 ± 2.3 |  | 43.6 ± 1.7 |  | 44.9 ± 1.5 |  | 0.226 |
| Sum C38 | 20.5 ± 4.0 | ^a^ | 18.5 ± 2.8 | ^ab^ | 15.8 ± 2.7 | ^b^ | 0.003 |
| Sum C40 | 2.87 ± 0.52 | ^a^ | 2.510 ± 0.410 | ^ab^ | 2.16 ± 0.37 | ^b^ | 0.001 |
|  |  |  |  |  |  |  |  |
| PE species |  |  |  |  |  |  |  |
| 32:0 | 0.10 ± 0.05 |  | 0.13 ± 0.06 |  | 0.14 ± 0.08 |  | 0.473 |
| 34:1 | 2.45 ± 1.09 |  | 2.76 ± 0.87 |  | 3.91 ± 1.67 |  | 0.253 |
| 34:2 | 6.40 ± 1.10 | ^b^ | 7.00 ± 0.91 | ^b^ | 8.76 ± 1.43 | ^a^ | <0.001 |
| 34:3 | 0.42 ± 0.11 | ^b^ | 0.43 ± 0.11 | ^b^ | 0.55 ± 0.14 | ^a^ | 0.015 |
| 36:2 | 19.0 ± 3.2 |  | 19.3 ± 2.1 |  | 21.4 ± 3.1 |  | 0.068 |
| 36:3 | 2.43 ± 0.60 | ^b^ | 2.47 ± 0.57 | ^b^ | 3.50 ± 0.73 | ^a^ | <0.001 |
| 36:4 | 8.47 ± 0.32 | ^b^ | 9.23 ± 0.66 | ^a^ | 9.16 ± 0.56 | ^a^ | 0.002 |
| 36:5 | 0.56 ± 0.09 |  | 0.57 ± 0.10 |  | 0.65 ± 0.09 |  | 0.054 |
| 38:1 | 0.09 ± 0.03 | ^a^ | 0.08 ± 0.03 | ^b^ | 0.11 ± 0.03 | ^a^ | 0.030 |
| 38:2 | 0.29 ± 0.03 |  | 0.29 ± 0.05 |  | 0.29 ± 0.05 |  | 0.950 |
| 38:4 | 40.1 ± 3.0 | ^a^ | 39.7 ± 3.4 | ^a^ | 35.3 ± 3.8 | ^b^ | 0.002 |
| 38:5 | 6.04 ± 0.74 |  | 6.01 ± 0.66 |  | 6.00 ± 0.82 |  | 0.420 |
| 38:6 | 6.82 ± 1.46 | ^a^ | 6.21 ± 1.10 | ^ab^ | 5.10 ± 0.98 | ^b^ | 0.003 |
| 40:4 | 1.20 ± 0.23 | ^a^ | 1.14 ± 0.14 | ^a^ | 0.92 ± 0.13 | ^b^ | 0.002 |
| 40:5 | 2.22 ± 0.48 | ^a^ | 1.97 ± 0.22 | ^ab^ | 1.78 ± 0.38 | ^b^ | 0.023 |
| 40:6 | 2.95 ± 0.63 | ^a^ | 2.40 ± 0.51 | ^ab^ | 2.05 ± 0.61 | ^b^ | 0.002 |
| 40:7 | 0.34 ± 0.14 |  | 0.28 ± 0.09 |  | 0.32 ± 0.15 |  | 0.537 |
| 40:8 | 0.12 ± 0.07 | ^a^ | 0.08 ± 0.04 | ^ab^ | 0.06 ± 0.03 | ^b^ | 0.023 |
|  |  |  |  |  |  |  |  |
| Sum C32 | 0.10 ± 0.05 |  | 0.13 ± 0.06 |  | 0.14 ± 0.08 |  | 0.473 |
| Sum C34 | 9.27 ± 2.20 |  | 10.2 ± 1.8 |  | 13.2 ± 3.1 |  | 0.737 |
| Sum C36 | 30.5 ± 3.5 | ^b^ | 31.5 ± 2.5 | ^b^ | 34.7 ± 3.2 | ^a^ | 0.004 |
| Sum C38 | 53.3 ± 4.5 | ^a^ | 52.3 ± 3.6 | ^a^ | 46.8 ± 5.2 | ^b^ | 0.001 |
| Sum C40 | 6.83 ± 1.28 | ^a^ | 5.87 ± 0.76 | ^ab^ | 5.13 ± 1.19 | ^b^ | 0.002 |

Data are means ± SD, *n* = 12–14 broilers/group

**Table S8** Individual lipid species composition of cholesteryl esters (CE), phosphatidylcholine (PC) and triacylglycerols (TG) in plasma of broilers fed diets with either 0% (HI-0), 2.5% (HI-2.5) or 5.0% (HI-5.0) *Hermetia illucens* (HI) larvae fat for 35 d

| **Fatty acid, % of total species** | **HI-0** |  | **HI-2.5** |  | **HI-5.0** |  | ***P*-value** |
| --- | --- | --- | --- | --- | --- | --- | --- |
| CE species |  |  |  |  |  |  |  |
| 14:0 | 0.44 ± 0.04 | ^c^ | 2.00 ± 0.19 | ^b^ | 3.63 ± 0.30 | ^a^ | <0.001 |
| 14:1 | 0.01 ± 0.01 | ^c^ | 0.05 ± 0.02 | ^b^ | 0.18 ± 0.03 | ^a^ | <0.001 |
| 15:0 | 0.10 ± 0.02 | ^c^ | 0.18 ± 0.03 | ^b^ | 0.26 ± 0.04 | ^a^ | <0.001 |
| 15:1 | 0.01 ± 0.01 | ^b^ | 0.01 ± 0.01 | ^b^ | 0.02 ± 0.01 | ^a^ | <0.001 |
| 16:0 | 19.0 ± 1.1 | ^ab^ | 19.4 ± 1.1 | ^a^ | 18.0 ± 0.9 | ^b^ | 0.003 |
| 16:1 | 2.64 ± 0.57 | ^b^ | 2.99 ± 0.44 | ^b^ | 5.12 ± 0.78 | ^a^ | <0.001 |
| 18:1 | 14.2 ± 1.4 |  | 14.5 ± 2.3 |  | 15.9 ± 1.5 |  | 0.064 |
| 18:2 | 57.0 ± 1.2 | ^a^ | 54.8 ± 2.2 | ^b^ | 52.0 ± 1.4 | ^c^ | <0.001 |
| 18:3 | 2.82 ± 0.21 | ^a^ | 2.64 ± 0.32 | ^a^ | 2.41 ± 0.17 | ^b^ | 0.001 |
| 20:3 | 0.77 ± 0.09 | ^a^ | 0.71 ± 0.08 | ^ab^ | 0.68 ± 0.07 | ^b^ | 0.024 |
| 20:4 | 2.19 ± 0.36 | ^a^ | 1.96 ± 0.43 | ^a^ | 1.32 ± 0.35 | ^b^ | <0.001 |
| 20:5 | 0.31 ± 0.03 | ^a^ | 0.28 ± 0.04 | ^b^ | 0.23 ± 0.04 | ^c^ | <0.001 |
| 22:4 | 0.01 ± 0.01 | ^a^ | 0.01 ± 0.01 | ^a^ | 0.01 ± 0.01 | ^b^ | 0.013 |
| 22:5 | 0.09 ± 0.02 | ^a^ | 0.08 ± 0.01 | ^a^ | 0.06 ± 0.03 | ^b^ | 0.003 |
| 22:6 | 0.40 ± 0.08 | ^a^ | 0.38 ± 0.10 | ^a^ | 0.25 ± 0.07 | ^b^ | <0.001 |
|  |  |  |  |  |  |  |  |
| Sum C14 | 0.45 ± 0.04 | ^c^ | 2.05 ± 0.20 | ^b^ | 3.81 ± 0.32 | ^a^ | <0.001 |
| Sum C15 | 0.10 ± 0.02 | ^c^ | 0.19 ± 0.03 | ^b^ | 0.28 ± 0.04 | ^a^ | <0.001 |
| Sum C16 | 21.6 ± 0.9 | ^b^ | 22.4 ± 0.9 | ^ab^ | 23.1 ± 0.8 | ^a^ | 0.001 |
| Sum C18 | 74.1 ± 1.2 | ^a^ | 72.0 ± 1.3 | ^b^ | 70.3 ± 1.0 | ^c^ | <0.001 |
| Sum C20 | 3.27 ± 0.33 | ^a^ | 2.95 ± 0.46 | ^a^ | 2.23 ± 0.41 | ^b^ | <0.001 |
| Sum C22 | 0.49 ± 0.09 | ^a^ | 0.46 ± 0.11 | ^a^ | 0.31 ± 0.10 | ^b^ | <0.001 |
|  |  |  |  |  |  |  |  |
| PC species |  |  |  |  |  |  |  |
| 30:0 | 0.03 ± 0.01 | ^c^ | 0.19 ± 0.03 | ^b^ | 0.35 ± 0.08 | ^a^ | <0.001 |
| 32:0 | 1.22 ± 0.14 |  | 1.38 ± 0.20 |  | 1.36 ± 0.18 |  | 0.054 |
| 32:1 | 0.41 ± 0.10 | ^c^ | 0.63 ± 0.13 | ^b^ | 1.15 ± 0.24 | ^a^ | <0.001 |
| 32:2 | 0.08 ± 0.01 | ^c^ | 0.39 ± 0.08 | ^b^ | 0.67 ± 0.27 | ^a^ | <0.001 |
| 34:1 | 9.06 ± 1.35 | ^b^ | 9.56 ± 1.63 | ^b^ | 11.5 ± 1.3 | ^a^ | <0.001 |
| 34:2 | 22.1 ± 2.3 |  | 24.4 ± 2.3 |  | 22.8 ± 3.8 |  | 0.062 |
| 34:3 | 0.87 ± 0.10 | ^b^ | 0.93 ± 0.12 | ^b^ | 1.04 ± 0.13 | ^a^ | 0.003 |
| 34:4 | 0.05 ± 0.01 | ^c^ | 0.09 ± 0.02 | ^b^ | 0.12 ± 0.03 | ^a^ | <0.001 |
| 36:1 | 3.44 ± 0.85 | ^b^ | 3.44 ± 0.94 | ^b^ | 5.10 ± 0.88 | ^a^ | <0.001 |
| 36:2 | 25.2 ± 2.1 |  | 24.8 ± 1.7 |  | 25.6 ± 1.6 |  | 0.494 |
| 36:3 | 4.68 ± 0.61 |  | 4.79 ± 0.41 |  | 4.99 ± 0.35 |  | 0.256 |
| 36:4 | 8.39 ± 1.31 | ^a^ | 7.92 ± 1.12 | ^a^ | 6.48 ± 1.20 | ^b^ | 0.001 |
| 36:5 | 0.65 ± 0.10 |  | 0.60 ± 0.08 |  | 0.57 ± 0.09 |  | 0.081 |
| 38:3 | 2.67 ± 0.45 | ^b^ | 2.55 ± 0.48 | ^b^ | 3.21 ± 0.42 | ^a^ | 0.001 |
| 38:4 | 13.6 ± 2.7 | ^a^ | 11.8 ± 2.4 | ^ab^ | 9.29 ± 3.12 | ^b^ | 0.002 |
| 38:5 | 2.94 ± 0.54 | ^a^ | 2.57 ± 0.33 | ^ab^ | 2.36 ± 0.57 | ^b^ | 0.017 |
| 38:6 | 1.97 ± 0.37 | ^a^ | 1.71 ± 0.32 | ^a^ | 1.32 ± 0.35 | ^b^ | <0.001 |
| 38:7 | 0.06 ± 0.02 | ^a^ | 0.04 ± 0.02 | ^b^ | 0.04 ± 0.02 | ^b^ | 0.006 |
| 40:4 | 0.58 ± 0.08 | ^a^ | 0.52 ± 0.07 | ^ab^ | 0.49 ± 0.08 | ^b^ | 0.025 |
| 40:5 | 0.93 ± 0.16 | ^a^ | 0.83 ± 0.12 | ^ab^ | 0.74 ± 0.18 | ^b^ | 0.021 |
| 40:6 | 0.91 ± 0.17 | ^a^ | 0.83 ± 0.19 | ^ab^ | 0.67 ± 0.21 | ^b^ | 0.009 |
| 40:7 | 0.15 ± 0.03 |  | 0.13 ± 0.02 |  | 0.13 ± 0.04 |  | 0.081 |
|  |  |  |  |  |  |  |  |
| Sum C30 | 0.03 ± 0.01 | ^c^ | 0.19 ± 0.03 | ^b^ | 0.35 ± 0.08 | ^a^ | <0.001 |
| Sum C32 | 1.71 ± 0.17 | ^c^ | 2.40 ± 0.31 | ^b^ | 3.18 ± 0.64 | ^a^ | <0.001 |
| Sum C34 | 32.0 ± 2.4 | ^a^ | 34.9 ± 2.2 | ^b^ | 35.5 ± 4.6 | ^ab^ | 0.011 |
| Sum C36 | 42.4 ± 1.8 |  | 41.5 ± 1.7 |  | 42.8 ± 1.7 |  | 0.193 |
| Sum C38 | 21.3 ± 3.3 | ^a^ | 18.6 ± 2.7 | ^ab^ | 16.2 ± 4.2 | ^b^ | 0.004 |
| Sum C40 | 2.57 ± 0.38 | ^a^ | 2.31 ± 0.36 | ^ab^ | 2.03 ± 0.48 | ^b^ | 0.010 |
|  |  |  |  |  |  |  |  |
| TG species |  |  |  |  |  |  |  |
| 42:0 | 0.01 ± 0.01 | ^c^ | 0.24 ± 0.15 | ^b^ | 0.89 ± 0.63 | ^a^ | <0.001 |
| 42:1 | n.d. |  | 0.60 ± 0.41 | ^a^ | 1.89 ± 1.54 | ^a^ | <0.001 |
| 42:2 | 0.01 ± 0.01 | ^b^ | 1.73 ± 0.88 | ^a^ | 3.95 ± 3.18 | ^a^ | <0.001 |
| 44:0 | 0.01 ± 0.01 | ^c^ | 0.45 ± 0.17 | ^b^ | 0.93 ± 0.56 | ^a^ | <0.001 |
| 44:1 | 0.01 ± 0.01 | ^c^ | 0.43 ± 0.20 | ^b^ | 1.24 ± 0.79 | ^a^ | <0.001 |
| 44:2 | n.d. |  | 0.72 ± 0.34 | ^a^ | 1.67 ± 1.27 | ^a^ | <0.001 |
| 46:0 | 0.04 ± 0.03 | ^c^ | 0.35 ± 0.12 | ^b^ | 0.59 ± 0.34 | ^a^ | <0.001 |
| 46:1 | 0.14 ± 0.08 | ^c^ | 1.4 ± 0.4 | ^b^ | 2.36 ± 0.98 | ^a^ | <0.001 |
| 46:2 | 0.01 ± 0.01 | ^b^ | 2.79 ± 1.06 | ^a^ | 3.72 ± 2.03 | ^a^ | <0.001 |
| 48:0 | 0.36 ± 0.21 | ^b^ | 0.54 ± 0.26 | ^ab^ | 0.75 ± 0.34 | ^a^ | 0.005 |
| 48:1 | 0.59 ± 0.25 | ^c^ | 1.87 ± 0.54 | ^b^ | 3.08 ± 1.08 | ^a^ | <0.001 |
| 48:2 | 0.38 ± 0.19 | ^b^ | 2.95 ± 0.76 | ^a^ | 3.75 ± 1.40 | ^a^ | <0.001 |
| 48:3 | 0.08 ± 0.05 | ^b^ | 3.86 ± 1.42 | ^a^ | 3.32 ± 1.71 | ^a^ | <0.001 |
| 49:1 | 0.10 ± 0.09 | ^c^ | 0.15 ± 0.05 | ^b^ | 0.23 ± 0.05 | ^a^ | <0.001 |
| 49:2 | 0.02 ± 0.02 | ^c^ | 0.15 ± 0.049 | ^b^ | 0.24 ± 0.10 | ^a^ | <0.001 |
| 49:3 | 0.01 ± 0.01 | ^b^ | 0.10 ± 0.05 | ^ab^ | 0.13 ± 0.09 | ^a^ | 0.007 |
| 50:1 | 3.44 ± 1.37 | ^b^ | 4.09 ± 1.21 | ^b^ | 5.40 ± 1.21 | ^a^ | 0.001 |
| 50:2 | 4.10 ± 1.14 | ^c^ | 5.33 ± 1.24 | ^b^ | 7.01 ± 0.70 | ^a^ | <0.001 |
| 50:3 | 1.47 ± 0.44 | ^c^ | 2.77 ± 0.38 | ^b^ | 3.49 ± 0.63 | ^a^ | <0.001 |
| 50:4 | 0.43 ± 0.143 | ^b^ | 1.31 ± 0.20 | ^a^ | 1.30 ± 0.21 | ^a^ | <0.001 |
| 50:5 | 0.08 ± 0.06 | ^b^ | 0.38 ± 0.06 | ^a^ | 0.35 ± 0.13 | ^a^ | <0.001 |
| 51:1 | 0.06 ± 0.04 | ^b^ | 0.13 ± 0.06 | ^a^ | 0.17 ± 0.07 | ^a^ | <0.001 |
| 51:2 | 0.13 ± 0.07 | ^c^ | 0.23 ± 0.05 | ^b^ | 0.29 ± 0.06 | ^a^ | <0.001 |
| 51:3 | 0.10 ± 0.05 | ^b^ | 0.17 ± 0.05 | ^a^ | 0.19 ± 0.04 | ^a^ | <0.001 |
| 51:4 | 0.11 ± 0.07 |  | 0.13 ± 0.05 |  | 0.11 ± 0.06 |  | 0.463 |
| 52:2 | 13.3 ± 3.2 |  | 13.9 ± 1.6 |  | 14.7 ± 2.7 |  | 0.582 |
| 52:3 | 14.6 ± 1.8 |  | 13.4 ± 2.2 |  | 11.6 ± 4.0 |  | 0.057 |
| 52:4 | 7.34 ± 1.40 | ^a^ | 4.86 ± 0.54 | ^b^ | 3.09 ± 0.94 | ^c^ | <0.001 |
| 52:5 | 2.21 ± 0.36 | ^a^ | 1.66 ± 0.13 | ^b^ | 1.31 ± 0.60 | ^c^ | <0.001 |
| 52:6 | 0.44 ± 0.16 | ^ab^ | 0.39 ± 0.04 | ^a^ | 0.35 ± 0.23 | ^b^ | 0.042 |
| 53:2 | 0.18 ± 0.08 | ^b^ | 0.27 ± 0.09 | ^a^ | 0.26 ± 0.08 | ^a^ | 0.022 |
| 53:3 | 0.23 ± 0.09 | ^ab^ | 0.27 ± 0.07 | ^a^ | 0.21 ± 0.05 | ^b^ | 0.036 |
| 53:4 | 0.22 ± 0.09 | ^a^ | 0.20 ± 0.06 | ^a^ | 0.11 ± 0.04 | ^b^ | <0.001 |
| 54:2 | 2.34 ± 0.69 |  | 2.28 ± 0.52 |  | 1.94 ± 0.49 |  | 0.174 |
| 54:3 | 5.92 ± 1.27 | ^a^ | 4.96 ± 1.17 | ^ab^ | 4.29 ± 1.68 | ^b^ | 0.021 |
| 54:4 | 9.18 ± 0.57 | ^a^ | 5.83 ± 0.85 | ^b^ | 4.06 ± 1.74 | ^c^ | <0.001 |
| 54:5 | 11.2 ± 2.2 | ^a^ | 5.91 ± 0.51 | ^b^ | 3.19 ± 1.21 | ^c^ | <0.001 |
| 54:6 | 8.50 ± 2.35 | ^a^ | 3.88 ± 0.31 | ^b^ | 1.73 ± 0.71 | ^c^ | <0.001 |
| 54:7 | 2.55 ± 0.71 | ^a^ | 1.07 ± 0.1 | ^b^ | 0.52 ± 0.32 | ^c^ | <0.001 |
| 56:4 | 0.85 ± 0.21 | ^a^ | 0.77 ± 0.23 | ^a^ | 0.51 ± 0.15 | ^b^ | <0.001 |
| 56:5 | 2.40 ± 1.29 |  | 2.23 ± 1.24 |  | 1.55 ± 0.77 |  | 0.128 |
| 56:6 | 2.91 ± 1.91 |  | 2.47 ± 1.55 |  | 1.72 ± 1.06 |  | 0.155 |
| 56:7 | 1.98 ± 1.43 |  | 1.45 ± 0.98 |  | 1.03 ± 0.79 |  | 0.114 |
| 56:8 | 1.05 ± 0.73 | ^a^ | 0.65 ± 0.40 | ^ab^ | 0.43 ± 0.40 | ^b^ | 0.023 |
| 58:7 | 0.47 ± 0.30 | ^a^ | 0.39 ± 0.26 | ^a^ | 0.21 ± 0.15 | ^b^ | 0.033 |
| 58:8 | 0.48 ± 0.32 | ^a^ | 0.38 ± 0.26 | ^a^ | 0.20 ± 0.18 | ^b^ | 0.015 |
|  |  |  |  |  |  |  |  |
| Sum C42 | 0.01 ± 0.01 | ^b^ | 2.57 ± 1.44 | ^a^ | 6.74 ± 5.33 | ^a^ | <0.001 |
| Sum C44 | 0.01 ± 0.01 | ^b^ | 1.6 ± 0.7 | ^a^ | 3.84 ± 2.59 | ^a^ | <0.001 |
| Sum C46 | 0.19 ± 0.07 | ^b^ | 4.53 ± 1.52 | ^a^ | 6.66 ± 3.31 | ^a^ | <0.001 |
| Sum C48 | 1.41 ± 0.66 | ^b^ | 9.21 ± 2.62 | ^a^ | 10.9 ± 4.4 | ^a^ | <0.001 |
| Sum C49 | 0.12 ± 0.08 | ^c^ | 0.40 ± 0.09 | ^b^ | 0.59 ± 0.21 | ^a^ | <0.001 |
| Sum C50 | 9.51 ± 2.74 | ^c^ | 13.9 ± 2.9 | ^b^ | 17.6 ± 1.6 | ^a^ | <0.001 |
| Sum C51 | 0.40 ± 0.21 | ^b^ | 0.66 ± 0.18 | ^a^ | 0.77 ± 0.17 | ^a^ | <0.001 |
| Sum C52 | 37.9 ± 3.0 | ^a^ | 34.1 ± 2.5 | ^b^ | 31.0 ± 7.2 | ^b^ | 0.002 |
| Sum C53 | 0.63 ± 0.23 |  | 0.74 ± 0.19 |  | 0.57 ± 0.13 |  | 0.096 |
| Sum C54 | 39.7 ± 4.0 | ^a^ | 23.9 ± 2.5 | ^b^ | 15.7 ± 5.6 | ^c^ | <0.001 |
| Sum C56 | 9.18 ± 5.36 |  | 7.57 ± 4.36 |  | 5.26 ± 3.09 |  | 0.063 |
| Sum C58 | 0.95 ± 0.61 | ^a^ | 0.77 ± 0.52 | ^a^ | 0.41 ± 0.32 | ^b^ | 0.015 |

Data are means ± SD, *n* = 12–14 broilers/group
